# Towards more fairness among states: How Dublin transfers redistribute asylum responsibility

**DOI:** 10.1177/14651165261446534

**Published:** 2026-05-13

**Authors:** Philipp Lutz, Florian Trauner, Philipp Stutz

**Affiliations:** 1 Department of Political Science and International Relations, 27212 University of Geneva, Geneva, Switzerland; 2 Department of Political Science and Public Administration, Vrije Universiteit Amsterdam, The Netherlands; 3 Brussels School of Governance, 70493 Vrije Universiteit Brussels, Brussels, Belgium

**Keywords:** Asylum, European Union, Dublin, responsibility-sharing, fairness

## Abstract

Solidarity and fairness in allocating refugee protection responsibilities is a central challenge in European asylum governance. The Dublin Regulation, assigning responsibility primarily to first countries of entry, has been criticised for exacerbating unequal burdens to member states. Yet systematic evidence on how Dublin transfers affect the distribution of responsibilities remains scarce. This article analyses Dublin statistics from 2008 to 2024 using a distribution key based on member states’ size and wealth as normative benchmark. We assess whether Dublin transfers reduce or exacerbate asymmetries between member states. Contrary to common perceptions, Dublin transfers modestly reduce inequitable distribution of asylum responsibilities, resulting from geographic arrival patterns paired with partial implementation. These findings challenge prevailing critiques and offer insights for evaluating the EU's solidarity mechanism.

## Introduction

Fairness among member states in the distribution of responsibilities for refugee protection has become a defining issue in European asylum governance. At the core of the Common European Asylum System (CEAS), the Dublin-III-Regulation has allocated responsibility for examining asylum claims primarily to the first EU member state an asylum seeker enters. EU policymakers, particularly those from member states at the external EU border, have repeatedly denounced the Dublin system as unjust because it places disproportionate responsibility on a few countries simply due to their geographic location (Fratzke, 2015). At the same time, the system has also drawn criticism from Central and Eastern European member states, who reject binding relocation quotas as unfair imposition on national sovereignty (Zaun, 2022). Northern destination countries, faced with significant onward movements of asylum seekers within the EU, have criticised the insufficient implementation of the ‘first country of entry’ rule and the limited effectiveness of Dublin transfers (Trauner, 2016). As a result, the Dublin system is broadly perceived as unfair across the EU, albeit for different reasons. Public opinion across Europe mirrors these divisions: many citizens have perceived the system as unfair and support stronger responsibility-sharing mechanisms (Bansak et al., 2017). Fairness in the distribution of refugees has thus become a central concern in both political debate and public attitudes towards EU asylum cooperation.

From June 2026 onwards, the Asylum and Migration Management Regulation (AMMR), as part of a new set of rules called the EU's New Pact on Migration and Asylum, replaces the Dublin-Regulation. Presented as a ‘historic’ breakthrough by senior EU officials (The Guardian, 2024), the reform package introduces a solidarity mechanism, obliging all member states to contribute to the collective management of asylum applications. Member states may choose between relocating asylum seekers and offering financial and operational support. However, critics argue that despite the new name, the substance of the Dublin system remains largely intact. Indeed, the key principle of assigning responsibility to the member state of first entry persists. Furthermore, the new Pact includes measures to tighten the compliance of member states (e.g., by converting ‘take-back requests’ into ‘take-back notifications’ and lengthening periods for remaining responsible for asylum applications), thereby potentially increasing the structural pressures on border states (ECRE, 2024; Statewatch, 2024).

Despite the prominence of the debate on fairness, systematic empirical assessments of how responsibility is actually distributed through the Dublin system remain scarce. Existing studies often document the broader asymmetry in asylum applications across member states (e.g., Takle and Seeberg, 2015; Vink and Meijerink, 2003) but fall short of analysing whether or how the Dublin mechanism itself contributes to, or mitigates, these imbalances. This article addresses this gap by examining to what extent the Dublin transfer system contributes to distributing responsibility fairly or unfairly among EU member states and what explains the observed outcomes. In doing so, we follow the EU's own terminology regarding ‘fairness mechanisms’ that should ensure a ‘fairer system based on solidarity’ through a ‘corrective allocation’ (European Commission, 2016), concepts we develop further in this article. Addressing this question is crucial, given that fairness has been repeatedly invoked as both a normative principle and a political demand in debates over reforming the CEAS and will continue to be a contentious issue under the reforms of the new Pact (Council of the EU, 2024; Trauner et al., 2025). Advancing a more transparent and evidence-based understanding of how member states behave within the Dublin framework is not only essential for assessing the system's fairness and effectiveness but also for informing the broader debate about solidarity and equitable responsibility-sharing in European asylum governance.

This article offers a new perspective on fairness among member states within the Dublin system by systematically analysing its actual operation over time. We begin by conceptualising and operationalising the fairness implications of the Dublin Regulation, focusing on how responsibility for asylum seekers is allocated and shared among member states. We then construct a new dataset from Eurostat statistics on requests and transfers between EU member states (2008–2024) to analyse how the Dublin system operates in practice. Our analysis assesses the extent to which the Dublin mechanism aligns with principles of equitable responsibility-sharing, allowing us not only to evaluate fairness at the aggregate system level but also to uncover variation in fairness-related behaviour across individual member states.

Our findings challenge conventional understandings of the Dublin system. The Dublin system has limited overall redistributive effects, but where transfers do affect the distribution, they modestly reduce asymmetries in asylum responsibilities between member states. We further demonstrate that this outcome can be explained by structural asymmetries in refugee arrivals and the partial compliance with the Dublin rules. These insights have important implications for our understanding of the functioning of the CEAS, the potential effectiveness of new solidarity mechanisms, and the broader normative foundations of asylum governance and fairness perceptions in the field of migration in the EU.

## Conceptualising ‘fairness’ in EU's asylum system

Responsibility-sharing lies at the core of international asylum governance. Within the CEAS, member states are expected to uphold the right to asylum collectively by distributing responsibilities in a manner that is both fair and sustainable. This expectation rests on normative and functional rationales: the legitimacy and effectiveness of common asylum systems depend not only on their operational performance but also on their ability to allocate responsibilities in ways perceived as fair.

Some conceptual clarifications are necessary to specify how this study understands and applies the notion of fairness in asylum governance. First, we focus on fairness in terms of *fairness to states*, defined as the equitable distribution of burdens and obligations among participating countries. This inter-state dimension is distinct from *fairness to refugees*, which concerns the rights and treatment of individuals within asylum procedures (Gibney, 2015; Ippolito and Velluti, 2011; Maiani, 2017). Although both dimensions matter for an overall evaluation of fairness in the system, they capture different actors and logics: distributive justice among states versus individual rights for protection-seeking migrants. It is therefore appropriate to analyse them separately. Second, fair responsibility-sharing can be implemented in different ways. It may involve the relocation of asylum seekers (people-sharing), but it can also consist of financial solidarity (money-sharing) to distribute costs more equitably, or policy harmonisation (norm-sharing) that ensures comparable asylum conditions across member states (Noll, 2003; Thielemann, 2018). In our discussion, we leave aside the question of financial and normative solidarity but focus on the distribution of asylum-seekers as the core dimension of responsibility-sharing. The physical distribution of people has proven particularly contentious politically: member states have shown fierce resistance to binding relocation quotas during the 2015–2016 crisis (Zaun, 2018). The new Pact had to introduce alternative contribution mechanisms (financial and operational support) alongside relocation to secure political agreement (De Bruycker, 2024). This shows that the question as to which state physically hosts asylum seekers remains the most divisive issue in EU asylum cooperation.

The concept of fairness in this realm has been approached from diverse disciplinary perspectives. From a legal viewpoint, the principle of solidarity and fair sharing of responsibility is anchored within EU law through Article 80 TFEU. Küçük (2016) argues that combining Article 80 with the Charter of Fundamental Rights raises constitutional questions about the fairness of the Dublin Regulation. Others highlight the coercive dynamics inherent in the Dublin system, where member states act defensively to protect national interests, thereby undermining cooperation (Dreyer-Plum, 2020; Maiani, 2016). Fairness thus emerges as both a foundational legal principle and a practical challenge, as the system's design can weaken the cooperative spirit that it seeks to uphold.

From a political science perspective, fairness is frequently analysed through the lens of collective action and public goods theory. Thielemann (2003) conceptualises EU asylum cooperation as a problem of international responsibility-sharing, with state behaviour influenced by either cost-benefit calculations or norms. This framework has been applied to the Dublin system and critical episodes like the 2015 European refugee crisis (Thielemann, 2018; Thielemann and Armstrong, 2013). The collective action framework illuminates why states sometimes accept disproportionate responsibilities and at other times engage in responsibility-shifting.

The notion of fairness to states has been conceptualised in various ways. A key question concerns the fairness principles and criteria to distribute responsibility for refugee protection across member states (see Lutz et al., 2021 for an overview; for a critique of quota allocation, see Maiani, 2017). Competing criteria have been proposed as bases for distribution keys, including population size, GDP, unemployment rate, territorial size and past efforts (Angeloni, 2019). These criteria build on different fairness principles: some emphasise equality, seeking to equalise shares regardless of capacity; others focus on equity by referring to distributing shares proportionally based on relevant capacities; still others draw on notions of merit or historical responsibility. These different principles may have diverging implications for a fair distribution. A single perspective likely is insufficient to find universal endorsement as they reflect different normative reference points and alignment with national interests (Gan, 2025).

In this discussion, it is important to note that the Dublin system was not designed to implement explicit principles of fairness; responsibility-sharing was at best a secondary consideration in its original design (Den Heijer et al., 2016). Instead, its primary aim was to avoid the phenomena of ‘asylum shopping’ and ‘refugees in orbit’ (Fratzke, 2015). The former describes the practice of asylum seekers applying in multiple member states or moving to those perceived to offer better protection prospects; the latter refers to situations in which member states avoid taking responsibility for asylum applications, leaving refugees in a state of legal and administrative limbo. The Dublin system sought to establish a transparent and unambiguous mechanism for assigning responsibility for an asylum seeker to a single participating state based on specific criteria (Guild et al., 2015). Over time, however, the system has faced growing criticism for disproportionately burdening member states at the EU external border, which are the primary entry points for most humanitarian migrants seeking to reach ‘Europe’ (Abrisketa Uriarte, 2022). Asylum seekers risk becoming stranded in countries with heavier burdens, such as Greece and Italy, contributing to deteriorating reception conditions and administrative bottlenecks. The core principle that responsibility lies with the state of first entry does not consider member states’ capacities or any proportional distribution logic. Consequently, the system is blamed for institutionalising asymmetry by placing heavier obligations on states at the EU's external border (Trauner, 2016).

Northern European states receiving fewer asylum seekers at their borders have been accused of using the Dublin Regulation as a tool for responsibility-shifting rather than responsibility-sharing (Fratzke, 2015). By enforcing transfers to the state of first entry, northern destination countries have sought to reduce their own caseloads, exacerbating tensions within the EU. Some of these states were however forced to suspend transfers to certain countries following court rulings that identified poor reception conditions in first-arrival countries, illustrating the system's contradictions (Moreno-Lax, 2012). The result is a fragmented and politically charged application of Dublin rules, where national interests often trump collective responsibility.

The absence of an embedded fairness logic remains therefore a persistent source of contestation. The Dublin system is widely perceived as failing to redistribute responsibility effectively across the EU (cf. Dreyer-Plum, 2020). Thus, and in response to the ‘migration crisis’ of 2015 and 2016, the Commission proposed ad hoc relocation schemes and to reform the Dublin system with a ‘corrective allocation mechanism’ to create a ‘fairer system based on solidarity’ (European Commission, 2016). Yet it was not until the New Pact on Migration and Asylum was adopted in 2024 that such a reform could be implemented, with a new solidarity mechanism providing an additional policy layer to address the system's imbalances. This indicates that issues of fairness have gained official recognition as policy priority, since leaving them unaddressed could further strain the system and undermine its legitimacy. Nevertheless, the political divide persists with some national governments remaining opposed to binding solidarity obligations (Holányi, 2025; Vaagland and Chmiel, 2024). Although fairness occupies a central place in political debates, and solidarity is an established norm in EU governance, fairness continues to generate political divisions, making systemic reform more challenging.

## Operationalising distributive fairness in the Dublin system

To evaluate the effects of the Dublin system on fairness, we rely on official Eurostat data covering requests, decisions, and transfers within the system, alongside the number of asylum applications submitted to each member state. We provide longitudinal statistical indicators spanning the period from 2008 to 2024.^1^ As far as we are aware, this represents the first systematic and longitudinal examination of Dublin cooperation. The data are of a directional dyadic structure with annual observations amounting to a sample size of N = 16,126 across 31 countries.^2^ This results in 930 directional dyads over 17 years encompassing a total of 564,062 Dublin transfers reported by member states (see Online Appendix for details on data preparation).^3^ However, the effective number of unique transfers is roughly half of this figure, as each transfer is typically reported by both the sending and receiving state. Due to discrepancies between these reports, the count based on outgoing transfers is 297,273, while the count based on incoming transfers is 266,789.^4^ This suggests that outgoing transfers are over-reported and/or incoming transfers are under-reported. We deal with this mismatch by using the sum of all reported transfers and use the two ways of reporting as robustness checks in the analysis.

While Eurostat data can be considered of rather high quality in terms of accessibility, validity and reliability, especially in comparison to individual country level data (Siruno et al., 2024: 8 and 19), it has some additional limitations. Most relevant for our purposes is that some countries report the number of Dublin requests rather than the number of persons, meaning an asylum seeker may be counted multiple times at EU level if present in several countries during the same year. Further measurement challenges include cross-country comparability in registration practices, some undercounting in certain member states, backlogs in data entry during peak periods (particularly 2015–2016), and comparability over time as states have joined or left the Dublin system (Siruno et al., 2024: 32–34). These limitations might affect the precision of transfer volumes but are not likely to bias our core findings based on directional patterns. Our main robustness check of comparing results using sending versus receiving country reports provides additional assurance that our core patterns are not resulting from measurement error.

In a first step, we calculate member states actual contributions to refugee protection in terms of the number of asylum applications they receive (see Online Appendix for the formal equations used in the operationalisation).^5^ As member states differ significantly in their capacity to host refugees, we need to set refugee arrivals into proportion to their capacity. For that purpose, we rely on the EU's own distribution key that operationalises fairness based on population size and the gross domestic product (GDP) (European Commission, 2015; Grech, 2016).^6^ It combines the egalitarian principle of population size with the capacity principle of countries’ wealth. This key was first presented in the Commission's proposal for a mandatory relocation quota in a Dublin-IV-regulation. While this proposal failed, the same key has found its way into the new Pact, concretely to define each member state's contribution to the Solidarity Pool (Article 66 of the Asylum and Migration Management Regulation, 2024/1351). We employ this benchmark as a reference to calculate how much member states may be expected to contribute to the overall number of asylum applications in the EU. This allows us to establish the extent to which countries’ numbers of asylum applications deviate from such an ideal-typical ‘fair’ distribution in a given year, yielding a continuous measure where values below 100% indicate under-fulfilment and values above 100% indicate over-fulfilment.^7^

Next, we analyse how Dublin transfers impact these asylum responsibilities of member states in the Dublin system. For this purpose, Dublin transfers are classified in terms of their fairness effects at two levels. First, we examine their effect on system-level asymmetry, focusing on whether a transfer contributes to a more or less fair distribution of asylum responsibilities across member states, or results in no change.^8^ Second, we examine dyad-level fairness, examining whether it makes the distribution of responsibilities between the two countries more or less fair, or leaves it unchanged. This two-tiered framework captures the dual nature of fairness in the Dublin system. At the system level, fairness concerns whether responsibility-sharing arrangements sustain the legitimacy and functionality of the collective governance framework. At the dyad level, fairness is experienced through concrete bilateral exchanges, shaping perceptions of justice, reciprocity, and trust between member states. Both dimensions are therefore central to understanding compliance, contestation, and support for reform.

At the system level, a transfer enhances fairness when it reallocates responsibility from a country exceeding its fair share to a country falling short of it. Conversely, a transfer reduces fairness if it shifts responsibility from a country below its fair share to one above it. Transfers that leave the overall distribution unchanged are considered distributionally neutral. This is the case when both the sending and the receiving state exceed their fair share (both >100%) or both fall short of it (both <100%). When assessing dyad-level fairness, a transfer is considered zero-sum (no redistributive effect) if it is offset by an equivalent transfer in the opposite direction. All remaining (non-reciprocal) transfers can then be evaluated as fair or unfair based on the two countries’ respective fair share fulfilment. A transfer is deemed fair if it reduces the gap between the two countries’ fair-share fulfilment rates. Conversely, it is unfair if it widens this gap.

The two concepts of distributive fairness are inherently connected yet analytically distinct. Dyad-level fairness concerns the relative balance of responsibility-sharing between two individual states, while system-level fairness refers to the aggregate redistributive effect across the entire Dublin system. A transfer that is dyadically fair (or unfair) may or may not alter system-level fairness, depending on whether it reduces or exacerbates overall distributional asymmetry. For instance, a transfer can improve fairness between two states while leaving the aggregate system unchanged, or it can worsen a bilateral relation without shifting the system balance. To capture these possibilities and because political contestation operates at both levels, transfers can be grouped into five distinct outcomes (Table 1).

**Table 1.** Relationship between system-level and dyad-level fairness of Dublin transfers.

Dyad-level fairness → System-level fairness ↓	Fair transfer *Net redistribution* *towards greater fairness*	Unfair transfer *Net redistribution* *towards lesser fairness*	Zero-sum *Equal in- and* *out-transfers*
Improves system fairness *Reduction in asymmetry*	(A) Fair at both levels *Responsibility-sharing*		
Worsens system fairness *Increase in asymmetry*		(B) Unfair at both levels *Responsibility-shifting*	
Neutral at system level *No change in asymmetry*	(C) Dyadically fair, but system-neutral	(D) Dyadically unfair, but system-neutral	(E) Zero-sum *Responsibility-swapping*

The combination of system-level and dyad-level fairness allows us to classify each Dublin transfer into one of five distinct fairness categories that are described in the following. First, a transfer can be *system-fair and dyad-fair* (A), simultaneously advancing fairness between two states and improving the overall balance of the system. For example, when a state exceeding its fair share transfers a case to a state that falls short of its fair share (as defined by our benchmark), the bilateral distribution becomes fairer and the system as a whole, moves closer to proportionality. These transfers contribute to a fairer European responsibility-sharing.

Second, transfers can be *system-unfair and dyad-unfair* (B), representing unfairness at both levels. Such transfers shift responsibility from a state already below its fair share to a state already exceeding it, thereby worsening bilateral fairness and pushing the system further away from a fair distribution.

Third, *system-neutral and dyad-fair* transfers (C) improve fairness between two states without altering overall distributive fairness. For example, if both states exceed their fair share and one shifts some responsibility to the other, the bilateral relationship may become more balanced, yet the aggregate asymmetry of the system remains unchanged.

Fourth, *system-neutral and dyad-unfair* transfers (D) reduce dyad-level fairness without affecting overall distributive fairness. If both states are below their fair share and one shifts asylum seekers to the other, the bilateral redistribution may be unfair, yet the system-level distribution remains unchanged as the transfer occurs entirely within the same side of the imbalance.

Finally, some transfers are *system-neutral and dyad-neutral* (E). Reciprocal exchanges of asylum seekers between two states cancel each other out, leaving both the system-level and dyad-level balance unchanged. As fairness is neither improved nor worsened, these transfers constitute a swapping of responsibilities with no redistributive effect.

Based on this conceptual framework, we then classify Dublin transfers using directional dyads in four steps. First, we decompose the number of transfers in directional dyads (MS1 → MS2 and MS2 → MS1) into *zero-sum transfers*, defined as the smaller of the two directional flows, and *net redistributive transfers*, defined as the absolute difference between the two flows. Only net transfers affect the distribution of asylum-seekers. Second, we classify each redistributive transfer as *dyadically fair* if it shifts responsibility from a country with a higher burden relative to its fair share to one with a lower burden, and as *dyadically unfair* if the transfer moves in the opposite direction. Third, transfers between two countries that are either both above or below their fair share are classified as *neutral at the system level*, as they do not improve overall fairness, even if they are fair or unfair at the dyad level. Fourth and as final step, we separate the dyadically fair/unfair transfers that are neutral at the system level, allowing us to correctly populate all five fairness categories. We can then aggregate the transfers at different levels to reveal how they contributed to fairness over time and across countries.

## Results

**Figure 1.**
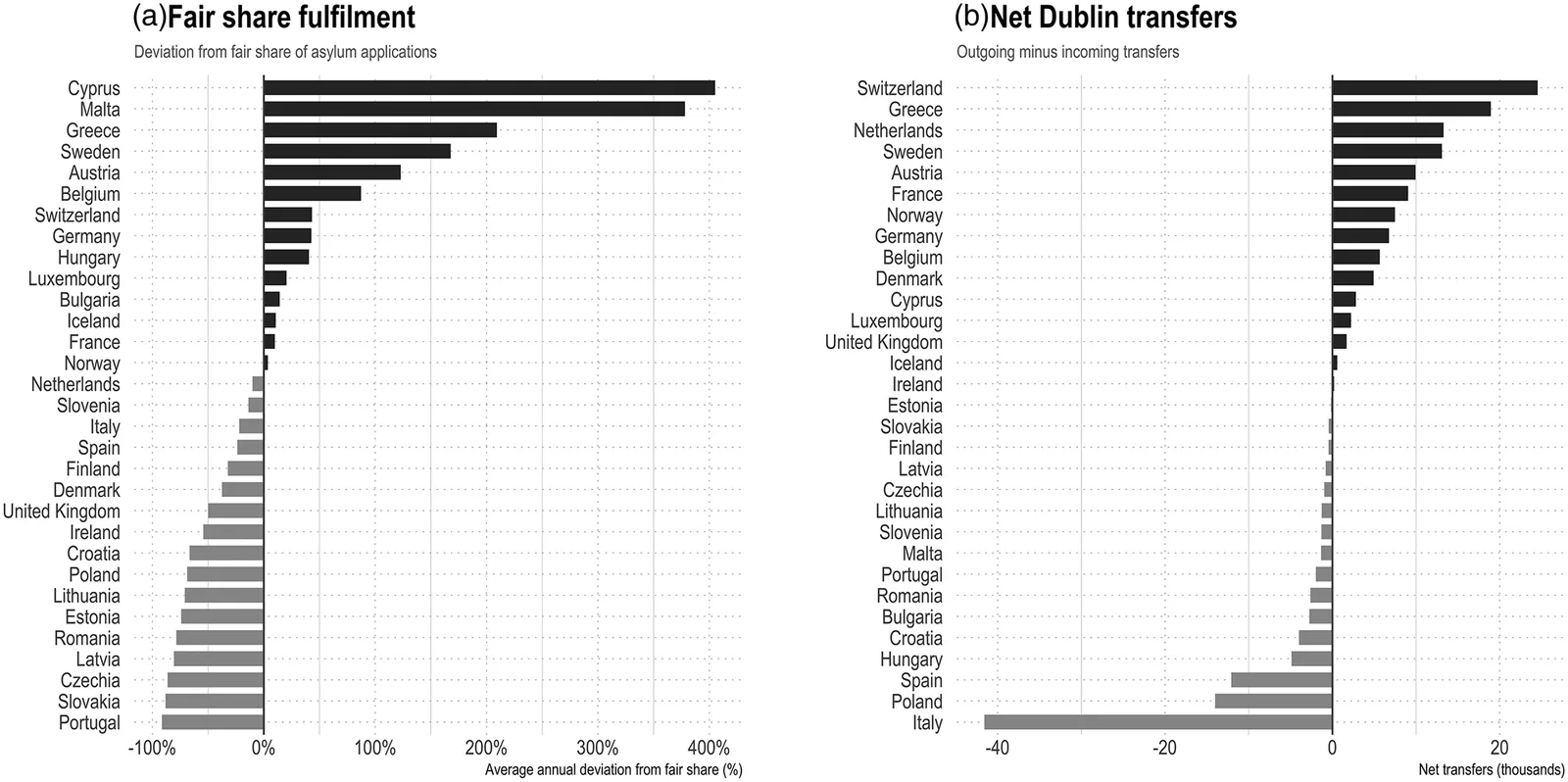
Distribution of fair share fulfilment and net transfers.

*Note:* Bar plot on the left showing the average deviation of countries’ number of asylum applications from the fair share based on the EU's distribution key in percentage points for the time period 2008–2024. Bar plot on the right showing the gap between outgoing and incoming transfers. Positive values mean that a member state transferred more people out than in (and vice versa). Calculation based on the statistical reporting of the respective member states. For the alternative calculation based on the partner countries’ statistical reporting see Online Appendix.

To establish the empirical baseline, we first examine countries’ relative asylum responsibilities, captured by their fair share fulfilment. Subsequently, we assess the extent to which Dublin transfers redistribute these responsibilities, measured by the net transfer rate. The measure of fair share fulfilment reveals two key insights about the Dublin system. First, the distribution of asylum seekers across member states is highly asymmetrical, lending empirical support to long-standing critiques of unfairness (Figure 1(a)). A clear geographic pattern emerges: southern countries at the EU's external borders and small Northern European destination countries tend to shoulder responsibilities well above their fair share, whereas many peripheral countries in Western and Eastern Europe assume substantially less. The second insight is that fair share fulfilment varies substantially over time (see Online Appendix). While some countries consistently exceed their fair share in terms of asylum responsibilities (e.g., Austria, Cyprus, Malta), others remain persistently below it (e.g., Croatia, Czech Republic, Estonia, Latvia, Lithuania, Poland, Portugal, Romania, and the United Kingdom). Most member states, however, experience considerable fluctuation, alternating between periods in which they receive more than their fair share of asylum applications and periods in which they fall short.

Next, the net transfer rate reveals the redistributive consequences of Dublin transfers (Figure 1(b)). For the studied period from 2008 to 2024, there is approximately the same number of countries which have more outgoing than incoming transfers and those which experience the reverse. The former group consists predominantly of Northern European destination countries, with Greece as a notable exception due to the judicially imposed suspension of incoming transfers from 2011 onwards, and Cyprus as more minor exception. The latter group consists mainly of countries on the southern and eastern periphery, which are more likely to be countries of first arrival. This pattern aligns with the prevailing narrative of the Dublin system as unfair, reflecting the perception that responsibility is shifted from wealthier northern states to already overburdened frontline countries in the EU's south.

**Figure 2.**
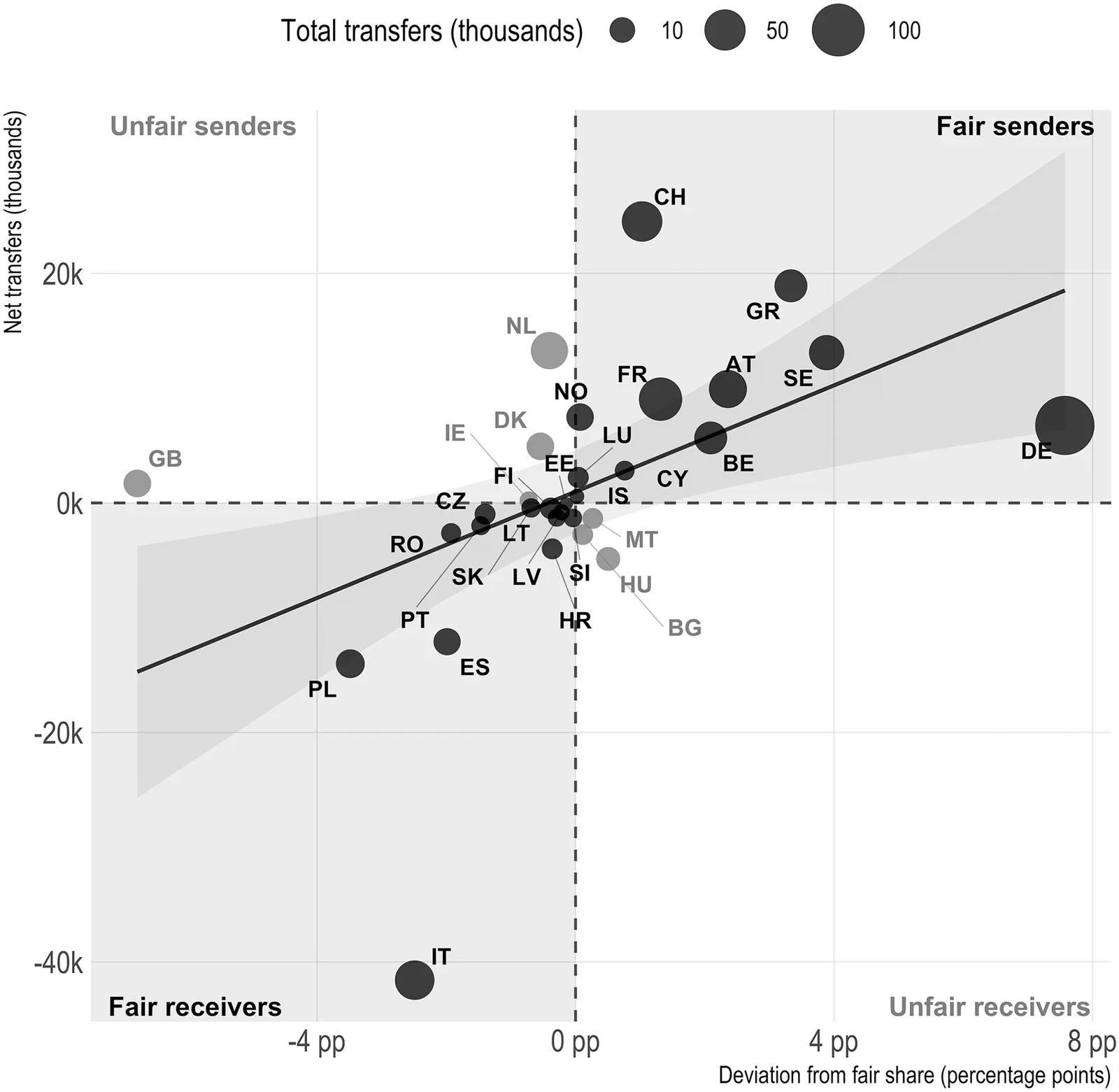
Scatter plot between countries’ fair share fulfilment and net transfers.

*Note:* Scatter plot between countries’ relative refugee exposure (Delta of actual share and fair share in percentage points) and their net Dublin transfers. A linear regression line with 95% confidence interval shows the bivariate association. Receivers have more incoming than outgoing transfers, and senders have more outgoing than incoming transfers. The size of the datapoints is scaled to the total number of transfers.

When we relate member states’ fair share fulfilment to their net Dublin transfers, a significant positive association emerges: countries receiving high numbers of asylum applications tend to carry out more outgoing transfers, while countries with lower numbers tend to receive more incoming transfers (Figure 2). This pattern aligns with the principle of fair and equitable responsibility-sharing. Most countries fall into the ‘fair’ quadrants, where the direction of their transfer balance corresponds appropriately to their relative exposure to the arrivals of asylum seekers. Fair receivers are primarily countries in Eastern and Southern Europe that have either low arrival numbers or many secondary movements to other member states. By contrast, fair senders are primarily Northern destination countries receiving high numbers of asylum applications. Only seven countries fall into the ‘unfair’ quadrants, either sending out more transfers despite under-contributing which is the case for some Western European countries or receiving more transfers despite over-contributing, occurring in three South-Eastern European countries. This means that, for the large majority of member states, their aggregate transfers through the Dublin system resonate with an overall fair distribution of asylum responsibilities, at least over the long-term. Even countries classified as unfair senders or receivers fall only slightly into the unfair zones, positioning close to the boundaries with fair quadrants. This confirms a general tendency towards fair transfers in the Dublin system.

**Figure 3.**
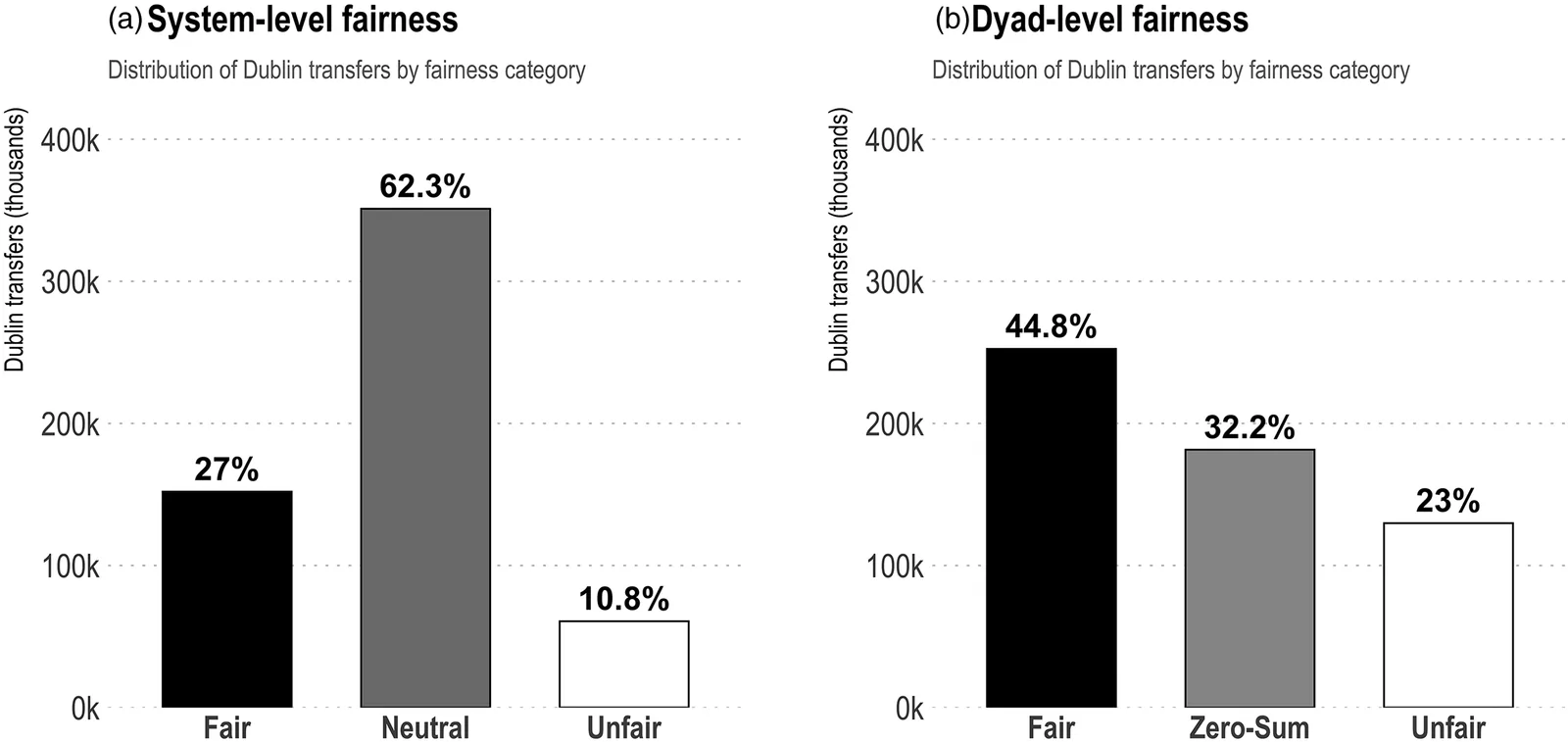
Dublin transfers by system-level and dyad-level fairness outcomes.

*Note:* Bar plots showing the number of transfers based on whether they contributed to more or less fairness, or have no effect on fairness, separately based on the system-level and dyad-level asymmetry in the distribution of asylum responsibilities. Transfers are thereby double counted (as each member state reports both incoming and outgoing transfers), allowing to balance out mismatching statistical reporting between them. For the absolute number of fair and unfair transfers by country, see Online Appendix.

However, this aggregate relationship does not yet speak directly to the fairness of the actual transfers. Fairness ultimately depends on the specific partner countries involved, as it matters *who* sends to or receives from *whom*. To assess what implications these transfers have for fairness, we classified them based on their implication for system fairness and fairness regarding the member state dyads (Figure 3). At the system level, more than six out of 10 transfers are neutral, meaning that they do not alter the asymmetry of the system. This dominant category is then followed by transfers that are classified as fair (27%), meaning that these are net transfers that go from a country above its fair share to a country below its fair share. The remaining 10.8% of transfers are the unfair redistributive transfers, resulting in a less fair distribution by countries.

These results indicate that Dublin transfers have only a limited impact on the overall distribution of asylum seekers across member states, though they show a modest tendency towards greater fairness. The pattern looks different for dyad-level fairness, as redistributive transfers are almost twice as frequent as at the system level. What is similar, however, is that fair transfers outnumber unfair ones by about two to one. In other words, while Dublin transfers are more redistributive in the dyad-level perspective than the system-level perspective, the relative fairness outcomes are largely similar. For robustness purposes, we re-calculate the fairness classification separately for outgoing transfers and incoming transfers to see whether reporting mismatches alter the result. There are some minor differences in the relative shares of fairness categories, but they do not significantly change the overall proportions (see Online Appendix).

We can then classify all five fairness categories (see Table 2). The difference here is that transfers that are fair or unfair dyadically can be neutral on the system level. While at the system level, neutral transfers clearly dominate. Transfers are more redistributive dyadically with twice as many classified as fair (44.8%) than as unfair (23%). The relative fairness is with 27% fair and 10.8% unfair transfers higher on the system level than on the dyad level. The pattern is, however, consistent in terms of responsibility-sharing outnumbering responsibility-shifting. Despite the significant share of fair transfers, most transfers within the Dublin system do not contribute in any way to a more desirable distribution of asylum-seekers between countries. This results from almost a third of transfers constituting a zero-sum swapping of responsibilities between member states.

**Table 2.** Number of Dublin transfers by fairness category.

Dyad-level fairness → System-Level fairness ↓	Fair transfer *Net redistribution* *towards greater fairness*	Unfair transfer *Net redistribution towards lesser fairness*	Zero-sum *Equal in- and out-transfers*	*Marginal distribution*
Improves system fairness *Reduction in asymmetry*	151,938 (27%)			151,938 (27%)
Worsens system fairness *Increase in asymmetry*		60,715 (10.8%)		60,715 (10.8%)
Neutral at system level *No change in asymmetry*	100,517 (17.8%)	69,061 (12.3%)	181,512 (32.2%)	351,090 (62.3%)
*Marginal distribution*	252,455 (44.8%)	129,776 (23%)	181,512 (32.2%)	563,743 (100%)

*Note:* Same as Table 1 but with the absolute and relative number of transfers that fall into the five fairness categories plus the marginal distributions for system-level and dyad-level fairness (percentages rounded to one decimal place). Aggregates based on 31 countries from 2008 to 2024.

**Figure 4.**
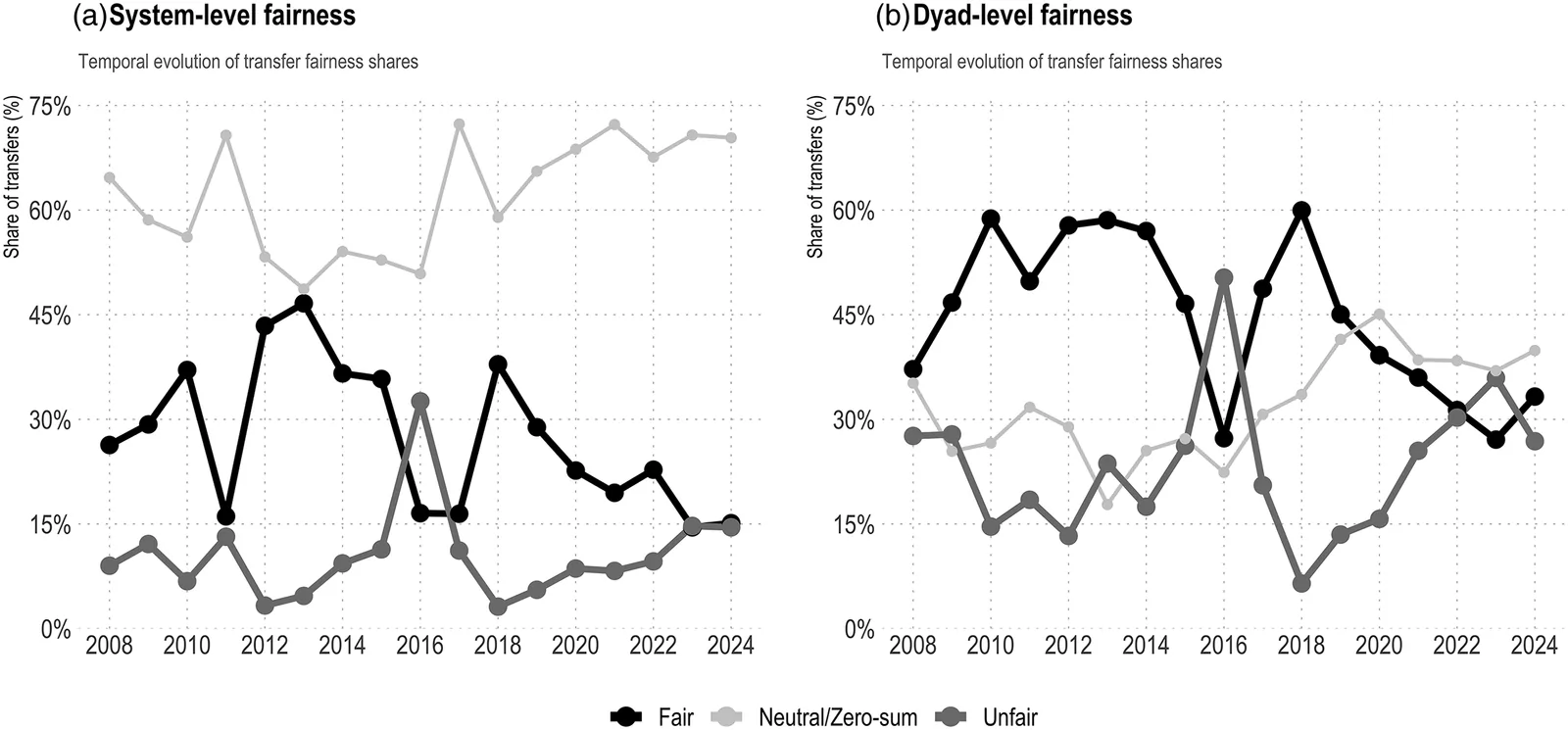
Redistributive Dublin transfers over time.

*Note:* Line plots showing the temporal evolution in the share of transfers based on whether they are offset by transfers in the reverse direction and whether they contributed to more (or less) fairness, separately based on the system-level and dyad-level asymmetry in the distribution of asylum responsibilities. For the fairness evolution by member state see Online Appendix.

In the next step, we look at how the fairness contributions have evolved over time (Figure 4). At the system level, we observe some variation over the years but there is no consistent trend. The relative importance of the three types of transfers remains largely stable over time. There is one exception: a clear effect of the 2015/2016 refugee crisis. In the second crisis year, the unfair transfers clearly outnumbered the fair transfers. This shift can be explained by higher fair share fulfilment in frontline countries resulting in transfers to these countries as unfair. This result is important as it suggests that the Dublin regulation worsened the distributional asymmetry during crisis times rather than supporting overburdened countries when they needed it most.^9^ Notably, in the last two to three years of the period of analysis, the fair and unfair transfers kept a balance, meaning that the Dublin system did not have any substantive redistributive effect. The fluctuation in the fairness-classification becomes even more pronounced when calculated separately for each member state (see Online Appendix), suggesting neither stable equilibrium nor a common trend.

**Figure 5.**
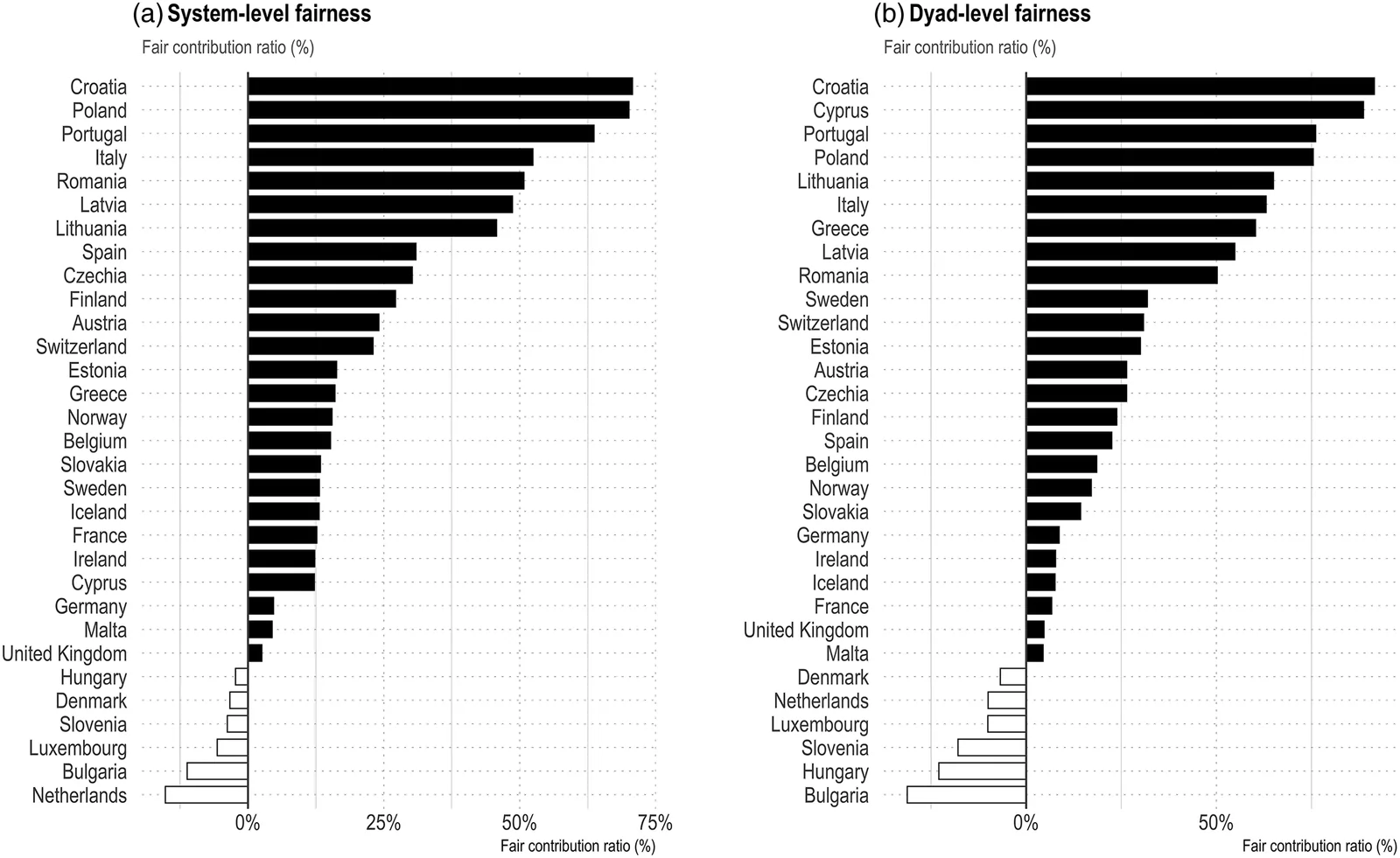
Countries’ redistributive transfers by relative fairness contribution.

*Note:* Bar plots showing the fair contribution ratio of member states between 2008 and 2024. The ratio is calculated as the share of fair redistributive transfers minus unfair redistributive transfers, divided by the total number of transfers. Positive values (black bars) indicate a net contribution to fairer distribution; negative values (white bars) indicate a net contribution to greater asymmetry. Countries are ordered in descending order. Aggregation based on the statistical figures provided by the reporting country. For a depiction as choropleth see Online Appendix.

Finally, we can also look at the fairness contribution of each member state and the extent to which their Dublin transfers share, shift or swap asylum responsibilities (Figure 5). Almost all countries contribute to more fairness in the Dublin system. This is remarkable and in line with the previous results. Only the Netherlands and Bulgaria contribute to greater asymmetry. The overall geographic pattern is that peripheral member states at the EU's external border, and thus likely countries of first arrival, contribute more to system-level fairness than countries situated more in the geographical centre of the EU. This is also in part due to zero-sum transfers, what we may frame as responsibility-swapping, dominating particularly in large countries like Germany, France, and the United Kingdom, thereby limiting the magnitude of their contribution to a fair share. The patterns for system-level and dyad-level fairness largely resemble one another.

Due to mismatches in the statistical reporting of member states of the same dyad, these results might differ, if we aggregate the data not based on the reporting country but based on the partner country. And indeed, the fairness outcomes do slightly differ with some member states changing positions in the ranking but overall confirming the structural pattern (see Online Appendix). The reported results are thus robust to mismatching statistical reporting.

Substantively, these results counter and question to some extent the often-heard critique that the system is not contributing to a fair allocation of asylum seekers because Northern countries are shifting responsibility to already overburdened frontline countries (e.g., Guild et al., 2015; Maiani, 2017). This is not confirmed. Even if modest in magnitude in the context of all asylum applications, the fairness effect of Dublin transfers occurs systematically. It is of substantive relevance and robust to different methodological choices and theoretical interpretations.

## Explaining the fairness contribution of Dublin transfers

Having established that fair transfers outnumber unfair ones, we now turn to explaining this pattern that goes against the common perception of the Dublin system. We consider three potential mechanisms that could account for the prevalence of fair transfers, each operating at a different level: institutional constraints through legal suspensions; political dynamics through selective implementation of Dublin transfer requests; and structural non-compliance based on migratory routes. We examine and discuss each mechanism empirically to assess its contribution to the observed fairness outcomes.

### Legal suspensions of transfers

One possible explanation for the surprising finding regarding the overall fairness of Dublin transfers relates to the judicial suspension of transfers in certain member states. Over the past decade, national and supranational courts have repeatedly intervened to halt transfers to countries where asylum procedures or reception conditions were deemed to violate fundamental rights (ECRE, 2018; Moreno-Lax, 2012). The most prominent example concerns Greece, where the European Court of Human Rights (ECtHR) in *M.S.S. v. Belgium and Greece* (2011) and the Court of Justice of the EU (CJEU) in *N.S. v. United Kingdom* (2011) ruled that transfers under the Dublin system could not take place due to systemic deficiencies in the Greek asylum and reception systems. Similar suspensions were later applied to Italy, Hungary, Bulgaria, Croatia and Malta, along with temporary suspensions of Poland and Romania in early 2022 due to reception capacity constraints following the influx of Ukrainian refugees (see Online Appendix for a complete overview with legal bases).

These suspensions effectively halted significant portions of transfers into, but not out of the affected countries. Because such countries are typically first-arrival states with high levels of asylum applications, these judicial interventions are likely to have had an equalising effect on the distribution of asylum responsibilities. By blocking a large number of transfers that would have otherwise sent asylum seekers back to overburdened states, court decisions prevented many potentially unfair redistributions, thereby improving fairness outcomes of the Dublin system.

To test this, we re-estimate fairness shares only for dyad-years without suspensions. When suspension dyad-years are excluded, the share of zero-sum and neutral transfers increases as expected, given that suspensions suppress transfers in one direction and make these dyad-years unusually asymmetric and thus disproportionately redistributive. The share of fair transfers decreases somewhat and unfair transfers increase modestly, narrowing the fair-to-unfair ratio compared to the main analysis. Nevertheless, fair transfers continue to clearly outnumber unfair ones at both the system and dyad level. Thus, while suspensions modestly shape the balance of transfer types, they do not drive the overall fairness outcome.

### Selective use of the Dublin system

A second explanation builds on the understanding that states may selectively use the Dublin system. For a transfer to occur, a member state must first submit a request, which must then be accepted by the requested state and subsequently implemented. This multi-step process provides governments with considerable leeway in both the submission and approval of requests (Woeffray, 2023). Non-compliance with Dublin rules is widespread among both member states and asylum seekers (Lutz et al., 2020; Trauner, 2016). This results in only around 10% of requests leading to actual transfers.^10^

This selective implementation of the Dublin system likely reflects both strategic choice and structural constraint: states tend to pursue transfers that are politically acceptable, administratively feasible, and normatively defensible, rather than simply applying the rules as written (ECRE, 2018; Fratzke, 2015; Woeffray, 2023). Limited administrative capacity and domestic political incentives thus introduce selection effects that shape which transfers ultimately take place. It is plausible, for instance, that states are more willing to enforce transfers perceived as fair or legitimate (for instance as a request comes from a member state whose administrative capacities are known to be stretched), while avoiding those that appear politically sensitive or burdensome. This selective enforcement could help explain why fair transfers occur more frequently than unfair ones.

To empirically assess whether selection effects at different stages of the Dublin procedure explain the prevalence of fair transfers, we examine whether the high attrition rate between requests and actual transfers. This attrition, caused by non-acceptance or non-implementation, may systematically filter out unfair transfers. We simulate two counterfactual scenarios: one in which all requests, and another in which all positive decisions, result in actual transfers. The resulting distributions are broadly similar to the observed ones. The share of fair transfers slightly increases, and unfair transfers slightly decrease (see Online Appendix). Analysing requests and decisions based on Eurodac fingerprint matches yields similar results (see Online Appendix). This suggests that inefficient or selective implementation has a modest negative effect on fairness, but does not fundamentally alter the picture, and does not explain the dominance of fair transfers. We also find no systematic relationship between countries’ fair-share fulfilment and their use of the sovereignty clause (Article 17 of the Dublin III Regulation) as a voluntary responsibility-sharing mechanism (Spearman ρ = −0.06, see Online Appendix), suggesting this discretionary tool does not meaningfully contribute to the fairness pattern we observe. The distributive effect of Dublin transfers does thus not result from selective use of the system by member states.

### Geography and migratory routes

A third explanation is structural, rooted in the geography of migration and asylum seekers’ destination preferences within Europe. Under Dublin rules, the country of first entry, whether through irregular border crossing or legal entry followed by visa overstay, is formally responsible for examining asylum claims. However, widespread non-compliance with this principle occurs through secondary movements: asylum seekers do not remain in their country of first arrival but instead move onward towards preferred destination countries in Northern and Western Europe, such as Germany or Sweden. This pattern of secondary movement prompts destination countries to issue transfer requests back to first-entry states.

This non-compliance by both asylum seekers pursuing better prospects and frontline states facilitating onward movement to reduce their own responsibilities (Lutz et al., 2020) creates a distinctive geographic pattern. Some of the highest numbers of asylum applications are recorded in Northern destination countries, which therefore tend to exceed their fair share, while many first-entry states at the EU external border fall below theirs. As a result, the structural dynamics of asylum seeker mobility and destination preferences may produce a pattern of transfers that aligns with a fairer overall distribution of responsibility across member states.

**Figure 6.**
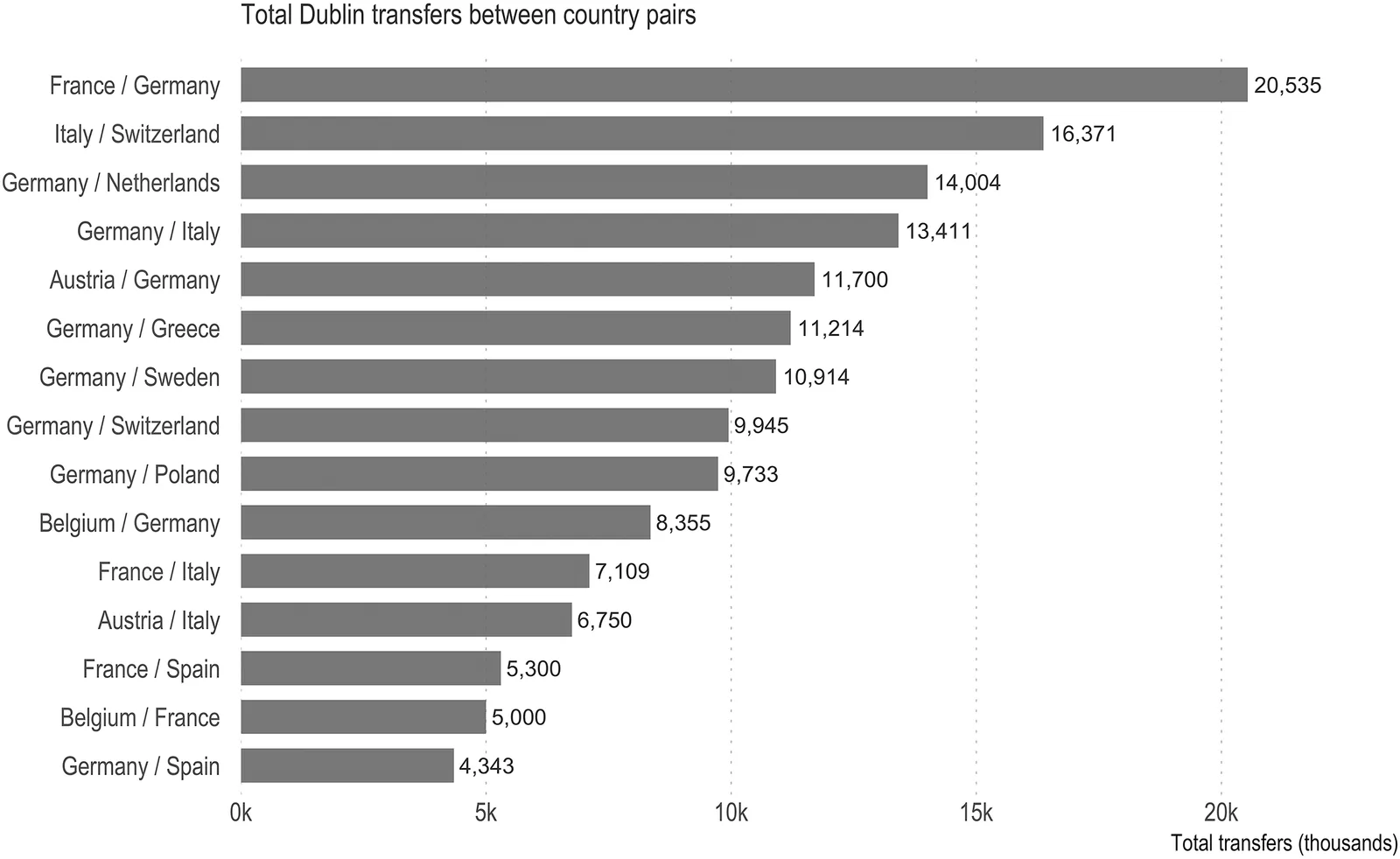
Country dyads with highest number of transfers.

*Note:* The graph shows the 15 dyads of EU member states with the highest total Dublin asylum-seeker transfers from 2008 to 2024. Transfers in both directions within each dyad have been aggregated. In case of mismatching statistical reporting between the member states in a dyad, the larger figure has been selected. Dyads are displayed alphabetically.

To substantiate this argument, we examine the most frequent transfer dyads (Figure 6). These indeed align with major migratory routes: transfers predominantly flow from Northern to Southern countries. In terms of fair share fulfilment, Germany mostly over-contributes, while Italy mostly under-contributes (see Online Appendix). This means in consequence that transfers from Germany to Italy tend to improve fairness, a pattern not limited to this specific dyad: similar dynamics are observed for transfers from other Northern states (e.g., Switzerland, Sweden) to frontline countries such as Greece and Spain, indicating a systematic north-to-south redistributive tendency along migration routes. Most countries with net-incoming transfers in Eastern and Southern Europe fall thus into the category of ‘fair receivers’ (Figure 2). Given that this pattern appears in particular for dyads with a high number of transfers crucially contributes to a relatively high share of fair transfers in the Dublin system. In sum, these empirical observations support the structural explanation: the geography of asylum seeker movements and destination preferences produces transfer patterns that improve distributional fairness.

## Conclusion

This article set out to examine how the Dublin system shapes the fairness of responsibility-sharing among EU member states. Focusing on *fairness to states*, defined as the distribution of asylum responsibilities across countries, we systematically analysed the direction of all Dublin transfers between 2008 and 2024, distinguishing between fairness effects at the bilateral level and at the level of the EU-wide system. The transfers of asylum-seekers between member states have different fairness implications as they can lead to responsibility-sharing, responsibility-shifting and responsibility-swapping. Contrary to common perceptions, we find that Dublin transfers have overall not been an unfair unloading of asylum responsibilities from Northern or Central European member states towards their southern and eastern peers. Instead, most transfers of member states with redistributive impact have actually contributed to a modest improvement in fairness relative to a no-transfer baseline.

Paradoxically, this fairness outcome emerges despite and not because of Dublin's inherently unfair responsibility-allocation rules. It has not resulted from a deliberately designed responsibility-sharing mechanism, nor from effective policy implementation. Rather, it emerges as a byproduct of the geographically uneven pattern of asylum seekers’ arrivals and the widespread non-compliance with Dublin's first-arrival principle. As Trauner (2016) observed, full application of the rules would overburden the South, while non-application would overburden the North. The actual functioning of the system (as we show it in this article) has therefore represented a muddling-through between these undesirable extremes. We establish that Dublin transfers actually contributed to a fairer distribution of asylum seekers across member states. Crucially, this holds at both analytical levels: the system corrects aggregate distributional asymmetries while simultaneously reducing bilateral inequities between member states. The widespread perception that Dublin is unfair to states does thus not reflect the actual re-distributional consequences of Dublin transfers in practice. It rather relates to the perception that the underlying responsibility-allocation rules are unfair and reinforce structural asymmetries.

This has important implications for the debates on the further development of EU asylum policy and the implementation of the EU's Pact on Migration and Asylum. A comparable outcome to Dublin's inadvertent fairness is now envisaged with the ‘solidarity mechanism’ of the new Pact, set to take effect in June 2026. However, it remains to be seen whether the formalisation of member states’ solidarity contributions will lead to a genuinely fairer EU asylum policy overall, or whether the negotiation and clarification of these contributions will instead become politically divisive and stalled. In practice, a selective implementation of solidarity tools could very well create new opportunities for member states to shirk or shift responsibilities in the asylum field (Trauner et al., 2025).

More broadly, our findings highlight the practical value of systematic fairness classifications and greater transparency in tracking distributive effects of EU asylum rules. If partial implementation fosters fairness, a clear trade-off emerges between efforts to enforce the Dublin rules by preventing secondary movements and a fair system of responsibility transfers. Understanding these fairness implications can provide guidance for the supplementary solidarity measures under the New Pact and suggests that taking the redistributive effects of transfers into account may create a more predictable and equitable asylum policy.

While providing important new insights, our study also has some limitations. We focused exclusively on distributive fairness between states, leaving aside questions of fairness to asylum seekers as well as procedural fairness. As such, we do not claim to offer an assessment of the fairness of the Dublin system in a broader sense. Moreover, our findings cannot be directly extrapolated to the context of the new Pact on Asylum and Migration even if the EU's new rules ‘on allocation of responsibility are very close to the current [Dublin] system’ (ECRE, 2024). That said, we hope to have provided an analytical tool for assessing redistributive dynamics in European asylum governance that can inform both scholarly debate and policy design. Future research can build on this framework and identify both the drivers and consequences of fluctuating fairness outcomes in European asylum governance. Understanding why perceptions of fairness diverge from distributive outcomes warrants further investigation as this policy field is crucial for the EU's wider legitimacy and standing.

## Supplemental Material

sj-pdf-1-eup-10.1177_14651165261446534 - Supplemental material for Towards more fairness among states: How Dublin transfers redistribute asylum responsibility Supplemental material, sj-pdf-1-eup-10.1177_14651165261446534 for Towards more fairness among states: How Dublin transfers redistribute asylum responsibility by Philipp Lutz, Florian Trauner and Philipp Stutz in European Union Politics

sj-zip-2-eup-10.1177_14651165261446534 - Supplemental material for Towards more fairness among states: How Dublin transfers redistribute asylum responsibility Supplemental material, sj-zip-2-eup-10.1177_14651165261446534 for Towards more fairness among states: How Dublin transfers redistribute asylum responsibility by Philipp Lutz, Florian Trauner and Philipp Stutz in European Union Politics

sj-zip-3-eup-10.1177_14651165261446534 - Supplemental material for Towards more fairness among states: How Dublin transfers redistribute asylum responsibility Supplemental material, sj-zip-3-eup-10.1177_14651165261446534 for Towards more fairness among states: How Dublin transfers redistribute asylum responsibility by Philipp Lutz, Florian Trauner and Philipp Stutz in European Union Politics

## Notes

1. Dublin transfers also occurred before 2008, but harmonised and comparable statistical data are only available from that year onward.

2. The sample covers Northern destination countries (Austria, Belgium, Denmark, Finland, France, Germany, Iceland, Ireland, Luxembourg, Netherlands, Norway, Sweden, Switzerland, United Kingdom), Southern frontline countries (Cyprus, Italy, Malta, Greece, Portugal, Spain) and Eastern European countries (Bulgaria, Croatia, Czechia, Estonia, Hungary, Latvia, Lithuania, Poland, Romania, Slovakia, Slovenia). We exclude Liechtenstein due to missing data, a member state that is negligible in terms of transfer volume. The classification into these three regions based on distinct positions within the European asylum regime is used throughout the article in line with previous research (Lutz and Karstens, 2021). Dublin statistics from Eurostat are only available at annual resolution, thus not allowing for a more fine-grained fairness classification.

3. The number of directional dyads is reduced to 870 for the years 2008–2012 and 2021–2024. The reason is that Croatia only joined the Dublin system in 2013 and the United Kingdom left the system in 2020. The unbalanced dataset might distort some aggregate fairness measures. We do however observe no discernible break or structural shift at the time of Croatia’s entry and UK’s exit both in the system-level aggregate as well as their neighbouring countries, suggesting robust results towards this unbalance in the dyadic data structure.

4. The largest contributors to this mismatch are Italy (ca. 36% of total discrepancy), Germany (ca. 9%), Poland (ca. 7%), Spain (ca. 5%) and Greece (ca. 5%), based on calculations by the authors.

5. We use asylum applications rather than positive decisions because Dublin allocates *examination* responsibility – transfers occur before decisions are made to determine which state must process the claim.

6. The original distribution key proposed by the European Commission in 2015 also included the unemployment rate and the number of previous asylum intakes, each weighted at 10% in the formula (see Grech, 2016). The formula was later simplified to comprise only population size and GDP, weighted equally.

7. While different allocation mechanisms for relocation and (re-)distribution have been proposed over the years, we follow the distribution key as proposed in 2020 as it is more basic but contains the most important dimensions and allows for flexibility while at the same time ensuring support for member states under migratory pressure (European Commission, 2020: 11 and 75). It goes back to the proposal for a Dublin IV regulation that never materialised (European Commission, 2015: 13 and 88).

8. Alternative variance-based continuous measures would allow for further variation by weighing reductions of greater asymmetries more heavily. We opt for categorical classification for its intuitive interpretation and consistent logic across both analytical levels.

9. Temporary emergency relocation mechanisms introduced during crisis periods proved limited in scope and effectiveness. The European Court of Auditors found that these schemes ‘did not reach their targets and so did not effectively alleviate the pressure on the Greek and Italian asylum systems’ (ECA, 2019: 5). As Nieswandt (2025) shows, this limited effectiveness further weakened political support for more far-reaching and binding reforms of the Dublin system.

10. Authors’ calculation based on Eurostat statistics.
