# Supplementary material for: Towards more fairness among states: How Dublin transfers redistribute asylum responsibility
Source: Eur Union Polit. 2026 May 13;27(3):533–56. doi: 10.1177/14651165261446534 (PMC13506051; doi:10.1177/14651165261446534)
Supplement: Supplementary file 1 [file sj-pdf-1-eup-10.1177_14651165261446534.pdf]

Online Appendix

Online Appendix for: "Towards more fairness among states: How Dublin Transfers Redistribute Asylum Responsibility", forthcoming in *European Union Politics*.

Philipp Lutz
University of Geneva, Switzerland
Vrije Universiteit Amsterdam, Netherlands
philipp.lutz@unige.ch

Florian Trauner
Vrije Universiteit Brussels, Belgium
florian.trauner@vub.be

Philipp Stutz
Vrije Universiteit Brussels, Belgium
philipp.stutz@vub.be

Table of Contents

A. DOCUMENTATION OF DATA PREPARATION 2

A.1 Dublin statistics 2
A.2 Calculation of member states' fair share fulfilment 4
A.3 Calculation of system-level fairness classification 4
A.4 Calculation of dyad-level fairness classification 4

B. DUBLIN SUSPENSIONS 5

B.1: List of Dublin suspensions 5

C. ADDITIONAL ANALYSES 6

C.1: Net transfers by member state (PARTNER aggregation) 6
C.2: Pyramid plot of Dublin transfers by member state and fairness outcome 7
C.3: Member states' fair share fulfilment in asylum responsibilities over time 8
C.4: System-level fairness outcomes over time by member state (aggregation reporting country) 9
C.5: Dyad-level fairness outcomes over time by member state (aggregation reporting country) 10
C.6: System-level fairness outcomes over time by member state (aggregation partner country) 11
C.7: Dyad-level fairness outcomes over time by member state (aggregation partner country) 12
C.8: Choropleth of countries' fair contribution ratio (reporting country aggregation) 13
C.9: Choropleth of countries' fair contribution ratio (partner country aggregation) 13
C.10: Share of Dublin transfers by fairness outcome (outgoing vs. incoming statistics) 14
C.11: Share of Dublin transfers by fairness category excluding legal suspensions 14
C.12: Fairness simulation based on Dublin requests and positive decisions 15
C.13: Fairness simulation with Dublin requests and positive decisions (Eurodac hits) 15
C.14: Top 10 countries using the Sovereignty Clause, 2014-2024 16

A. Documentation of data preparation

A.1 Dublin statistics

The analysis of Dublin transfers is based on Eurostat's official Dublin statistics datasets (code migr_dub), specifically the following sub-datasets:

- migr_dubro — Outgoing requests under the Dublin Regulation
- migr_dubri — Incoming requests under the Dublin Regulation
- migr_dubroa — Outgoing transfers from the reporting Member State
- migr_dubria — Incoming transfers to the reporting Member State
- migr_dubdec — Decisions taken by Member States under the Dublin Regulation

Data were downloaded from Eurostat and merged into a harmonised panel by reporting country (GEO), partner country (PARTNER), and year. Certain aggregate entries (e.g., "European Union - 27 countries (from 2020)", "European Union - 28 countries (2013–2020)", and "Total") were excluded, as well as country-specific exclusions for Liechtenstein, Croatia (pre-2013), and United Kingdom (post-2020).

Missing values were identified for requests, transfers, and decisions. Where possible, missing entries were inferred from the corresponding reverse dyad (i.e., when GEO's incoming request was missing but the partner's outgoing request existed). Partial missing values for transfers and decisions were treated similarly to maximise coverage. Suspensions are shown in Table A1.

This filtering and cleaning process ensures that the final dataset is consistent, comparable across member states, and suitable for both system-level and dyad-level analyses.

The additional statistical data on Eurodac hits is based on the Eurostat datasets migr_dubredaco, migr_dubredaci, migr_dubdedaco and migr_dubdedaci.

Dataset references

Eurostat. (n.d.-a). "Incoming 'Dublin' Requests by Submitting Country (PARTNER), Type of Request, Legal Provision, Sex and Type of Applicant." Dataset MIGR_DUBRI. Accessed March 13, 2026. https://doi.org/10.2908/MIGR_DUBRI.

Eurostat. (n.d.-b). "Outgoing 'Dublin' Requests by Receiving Country (PARTNER), Type of Request, Legal Provision, Sex and Type of Applicant." Dataset MIGR_DUBRO. Accessed March 13, 2026. https://doi.org/10.2908/MIGR_DUBRO.

Eurostat. (n.d.-c). "Incoming 'Dublin' Requests Based on EURODAC by Submitting Country (PARTNER), Type of Request and Legal Provision." Dataset MIGR_DUBREDACI. Accessed March 13, 2026. https://doi.org/10.2908/MIGR_DUBREDACI.

Eurostat. (n.d.-d). "Outgoing 'Dublin' Requests Based on EURODAC by Receiving Country (PARTNER), Type of Request and Legal Provision." Dataset MIGR_DUBREDACO. Accessed March 13, 2026. https://doi.org/10.2908/MIGR_DUBREDACO.

Eurostat. (n.d.-e). "Decisions on Incoming 'Dublin' Requests by Submitting Country (PARTNER), Type of Decision, Type of Request, Legal Provision, Sex and Type of Applicant." Dataset MIGR_DUBDI. Accessed March 13, 2026. https://doi.org/10.2908/MIGR_DUBDI.

Eurostat. (n.d.-f). "Decisions on Outgoing 'Dublin' Requests by Receiving Country (PARTNER), Type of Decision, Type of Request, Legal Provision, Sex and Type of Applicant." Dataset MIGR_DUBDO. Accessed March 13, 2026. https://doi.org/10.2908/MIGR_DUBDO.

Eurostat. (n.d.-g). "Decisions on Incoming 'Dublin' Requests Based on EURODAC by Submitting Country (PARTNER), Type of Decision, Type of Request, Legal Provision, Sex and Type of Applicant." Dataset MIGR_DUBDEDACI. Accessed March 13, 2026. https://doi.org/10.2908/MIGR_DUBDEDACI.

Eurostat. (n.d.-h). "Decisions on Outgoing 'Dublin' Requests Based on EURODAC by Receiving Country (PARTNER), Type of Decision, Type of Request, Legal Provision, Sex and Type of Applicant." Dataset MIGR_DUBDEDACO. Accessed March 13, 2026. https://doi.org/10.2908/MIGR_DUBDEDACO.

Eurostat. (n.d.-i). "Incoming 'Dublin' Transfers by Submitting Country (PARTNER), Legal Provision, Duration of Transfer, Sex and Type of Applicant." Dataset MIGR_DUBTI. Accessed March 13, 2026. https://doi.org/10.2908/MIGR_DUBTI.

Eurostat. (n.d.-j). "Outgoing 'Dublin' Transfers by Receiving Country (PARTNER), Legal Provision, Duration of Transfer, Sex and Type of Applicant." Dataset MIGR_DUBTO. Accessed March 13, 2026. https://doi.org/10.2908/MIGR_DUBTO.

Eurostat. (n.d.-k). "Asylum Applicants by Type of Applicant, Citizenship, Age and Sex — Annual Aggregated Data." Dataset MIGR_ASYAPPCTZA. Accessed March 13, 2026. https://doi.org/10.2908/MIGR_ASYAPPCTZA.

World Bank. 2024a. "GDP (Current US$)." World Development Indicators. Dataset API_NY.GDP.MKTP.CD_DS2_en_excel_v2_783994. Washington, DC: World Bank. Accessed March 13, 2026. https://data.worldbank.org/indicator/NY.GDP.MKTP.CD.

World Bank. 2024b. "Population, Total." World Development Indicators. Dataset API_SP.POP.TOTL_DS2_en_excel_v2_784032. Washington, DC: World Bank. Accessed March 13, 2026. https://data.worldbank.org/indicator/SP.POP.TOTL.

A.2 Calculation of member states' fair share fulfilment

$$\text{Fair share fulfilment}_{i,t} = \left(\frac{\text{Applications}_{i,t}}{\sum_j \text{Applications}_{j,t}} \Bigg/ \frac{0.5 \cdot \text{Population}_{i,t} + 0.5 \cdot \text{GDP}_{i,t}}{\sum_j (0.5 \cdot \text{Population}_{j,t} + 0.5 \cdot \text{GDP}_{j,t})} \right) \times 100$$

A.3 Calculation of system-level fairness classification

Let $T_{i \to j}$ and $T_{j \to i}$ denote the number of transfers from country i to country j and vice versa.

Define the offset zero-sum transfers between i and j as:

$$Z_{ij} = \min(T_{i \to j}, T_{j \to i})$$

Let F_i and F_j denote the fair share fulfilment of countries i and j, where values greater than 100 indicate above and less than 100 indicate below the fair share.

Then, the classification of transfers is given by:

$$\text{Neutral transfers}_{ij} = Z_{ij} + \sum_{\substack{r,s \in \{i,j\} \\ r \neq s}} [T_{r \to s} - Z_{ij}] \cdot \mathbf{1}_{\{(F_r > 100 \wedge F_s > 100) \vee (F_r < 100 \wedge F_s < 100)\}}$$

$$\text{Fair transfers}_{ij} = \sum_{\substack{r,s \in \{i,j\} \\ r \neq s}} [T_{r \to s} - Z_{ij}] \cdot \mathbf{1}_{\{F_r > 100 \wedge F_s < 100\}}$$

$$\text{Unfair transfers}_{ij} = \sum_{\substack{r,s \in \{i,j\} \\ r \neq s}} [T_{r \to s} - Z_{ij}] \cdot \mathbf{1}_{\{F_r < 100 \wedge F_s > 100\}}$$

where $\mathbf{1}_{\{\cdot\}}$ is the indicator function equal to 1 if the condition holds and 0 otherwise.

A.4 Calculation of dyad-level fairness classification

Let $T_{i \to j}$ and $T_{j \to i}$ denote the number of transfers from country i to country j and vice versa.

Define the offset zero-sum transfers between i and j as:

$$Z_{ij} = \min(T_{i \to j}, T_{j \to i})$$

Let F_i and F_j denote the fair share fulfilment of countries i and j.

Then, the classification of transfers at the dyadic level is:

$$\text{Zero-sum transfers}_{ij} = Z_{ij}$$

$$\text{Fair transfers}_{ij} = (T_{i \to j} - Z_{ij}) \cdot \mathbf{1}_{\{F_i > F_j\}} + (T_{j \to i} - Z_{ij}) \cdot \mathbf{1}_{\{F_j > F_i\}}$$

$$\text{Unfair transfers}_{ij} = (T_{i \to j} - Z_{ij}) \cdot \mathbf{1}_{\{F_i < F_j\}} + (T_{j \to i} - Z_{ij}) \cdot \mathbf{1}_{\{F_j < F_i\}}$$

where $\mathbf{1}_{\{\cdot\}}$ is the indicator function equal to 1 if the condition holds and 0 otherwise.

B. Dublin suspensions

B.1: List of Dublin suspensions

Country	Transfer status	Legal basis	Key concerns	Period
Greece	Full suspension	ECtHR *M.S.S.* (2011),[a] CJEU *N.S.* (2011)[b]	Systemic deficiencies in asylum procedure and reception conditions	2011–present
Hungary	Full/widespread	CJEU *C-808/18* (2020),[c] national court decisions	Systemic violations, detention, non-refoulement risks	2015–present
Bulgaria	Partial/case-by-case	UNHCR recommendations (2014),[d] national courts	Reception conditions, vulnerability assessments, refoulement risks	2014–present
Italy	Intermittent/partial	ECtHR *Tarakhel* (2014),[e] official suspension (Dec 2022)[f]	Reception capacity, vulnerable persons requiring guarantees	2014–present
Croatia	Partial (multiple courts)	National court decisions[g]	Pushback allegations, chain refoulement risks	2022–present
Malta	Sporadic court suspensions	ECtHR *Feilazoo* (2021),[h] national courts	Detention conditions and duration	2021–present
Poland	Temporary suspension	Capacity constraints (Ukraine)[i]	Reception system capacity	Feb–Aug 2022
Romania	Temporary suspension	Capacity constraints (Ukraine)[i]	Reception system capacity	Early 2022

[a] ECtHR, *M.S.S. v. Belgium and Greece*, Application no. 30696/09, 21 January 2011.
[b] CJEU, Joined Cases C-411/10 and C-493/10, *N.S. v. Secretary of State for the Home Department*, 21 December 2011.
[c] CJEU, Case C-808/18, *Commission v. Hungary*, 17 December 2020.
[d] UNHCR, *Observations on the current situation of asylum in Bulgaria*, January 2014.
[e] ECtHR, *Tarakhel v. Switzerland*, Application no. 29217/12, 4 November 2014.
[f] Italian Dublin Unit, Letter to Dublin Units, 5 December 2022 (cited in ECRE, 2023).
[g] Netherlands Council of State, ECLI:NL:RVS:2022:1042, 13 April 2022; Belgium CALL, Decision 281 237, 5 December 2022.
[h] ECtHR, *Feilazoo v. Malta*, Application no. 6865/19, 11 June 2021.
[i] ECRE (2023), *The Implementation of the Dublin III Regulation in 2022*, pp. 23–27.

C. Additional analyses

C.1: Net transfers by member state (PARTNER aggregation)

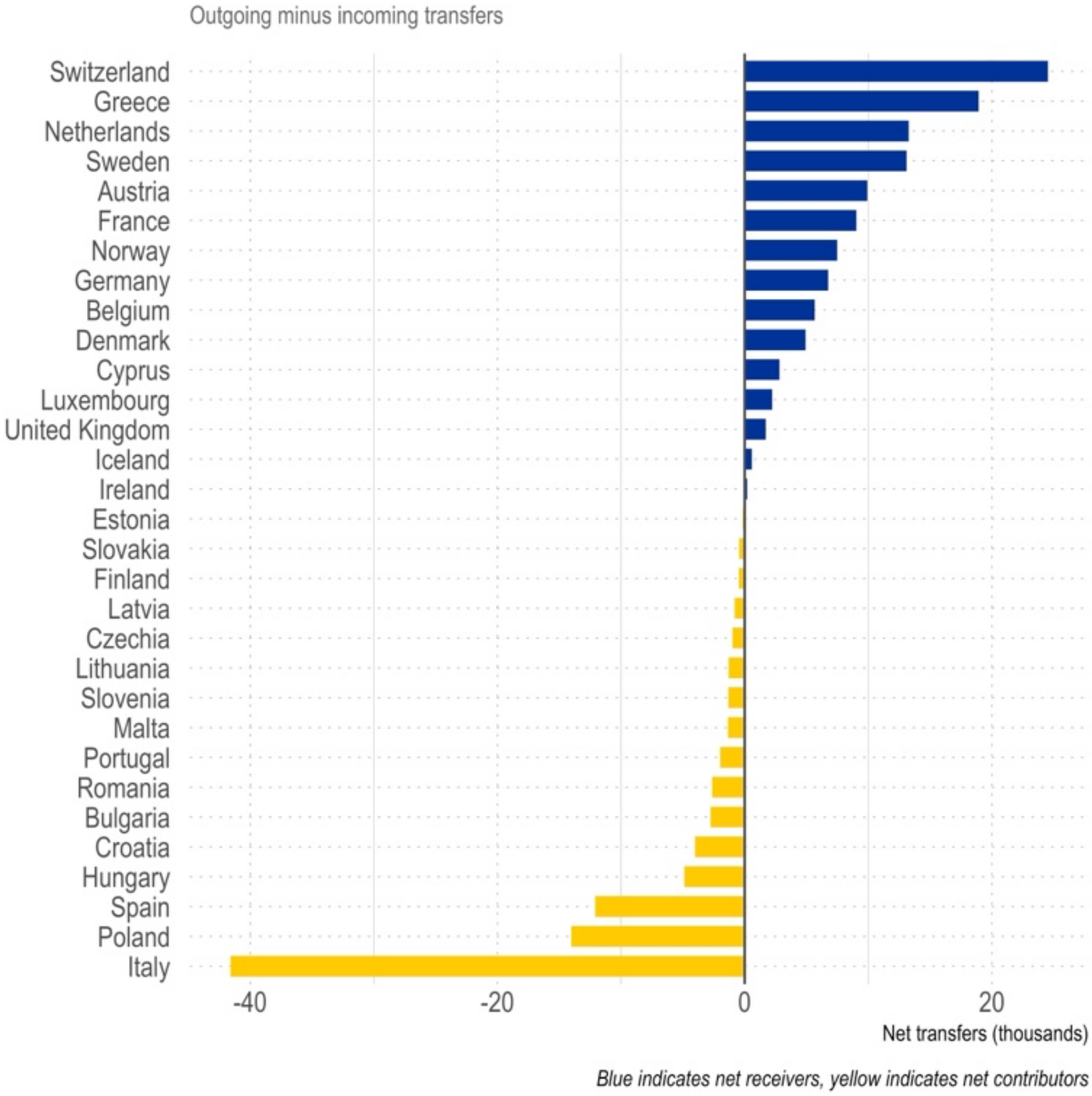

Note: Same plot as Figure 1 in the main manuscript with the difference that the calculation is based on the reported transfers of the partner country.

C.2: Pyramid plot of Dublin transfers by member state and fairness outcome

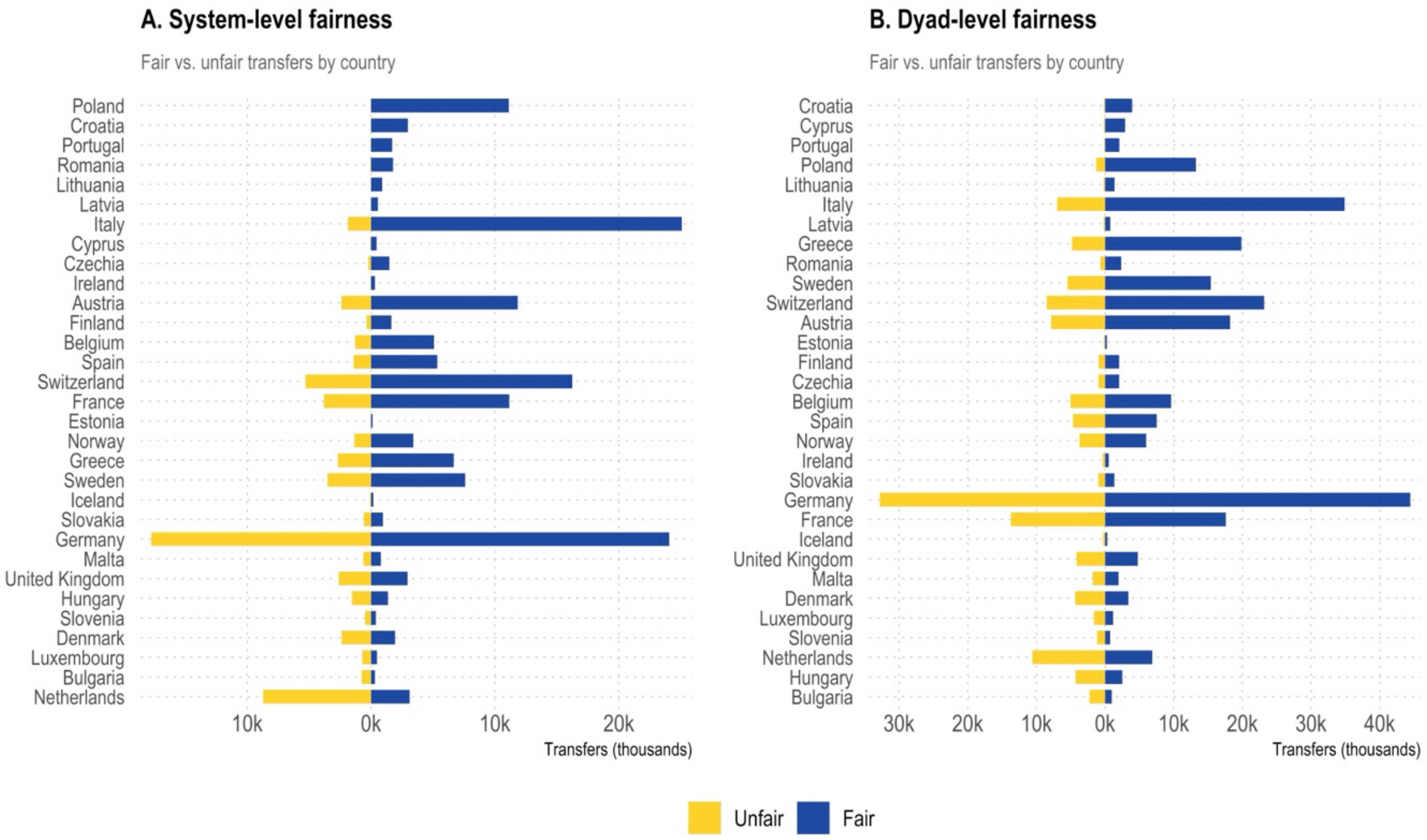

Note: Pyramid plots showing the absolute number of Dublin transfers per country (both incoming and outgoing) that are classified as fair and unfair on a system-level and within dyads. Countries are sorted in descending order of the ratio of fair to unfair transfers.

C.3: Member states’ fair share fulfilment in asylum responsibilities over time

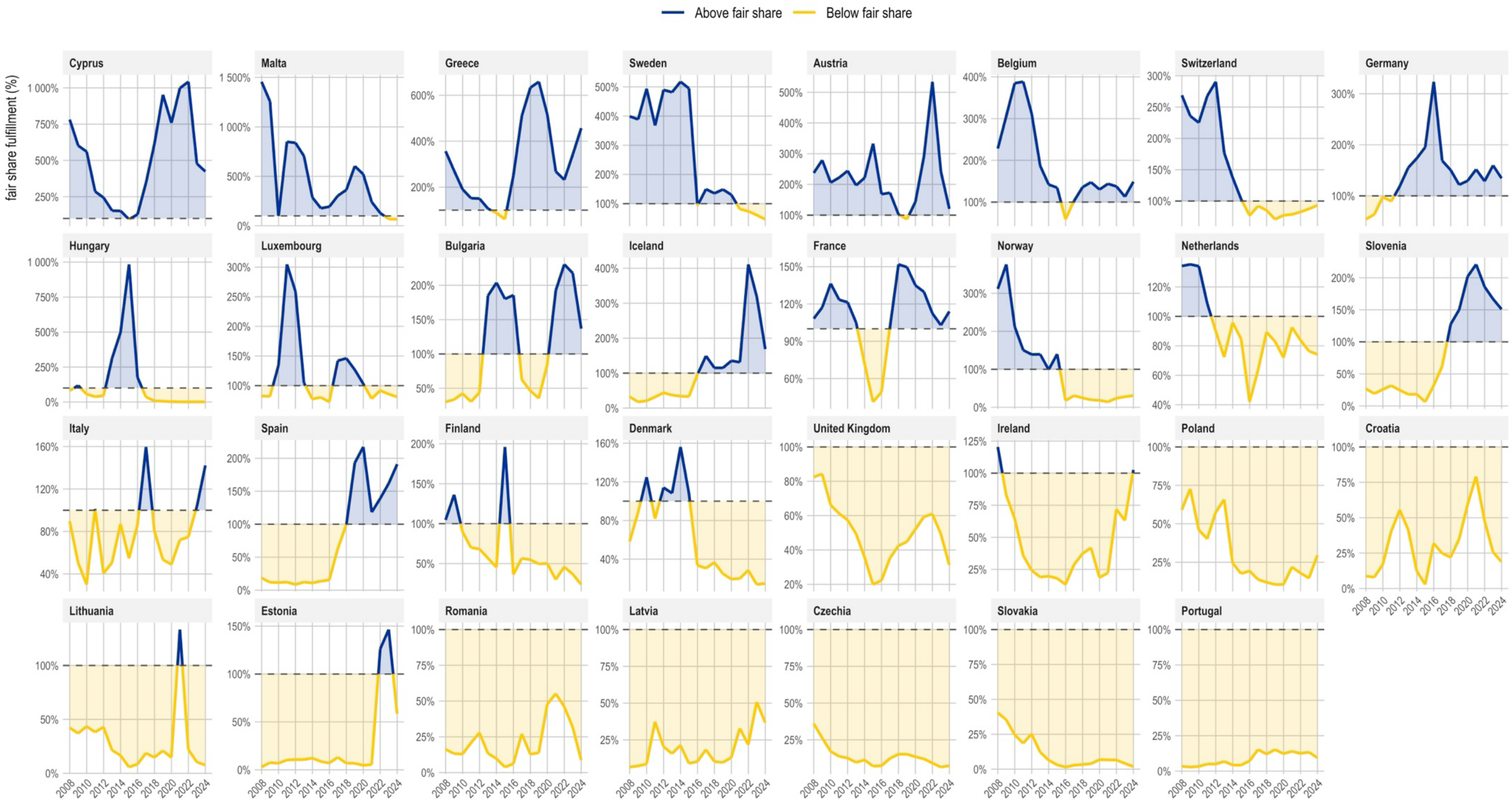

Note: Line plots of countries’ fair share fulfilment over time, sorted by countries’ mean fair share fulfilment in descending order. Values above fair share in blue and below in yellow.

C.4: System-level fairness outcomes over time by member state (aggregation reporting country)

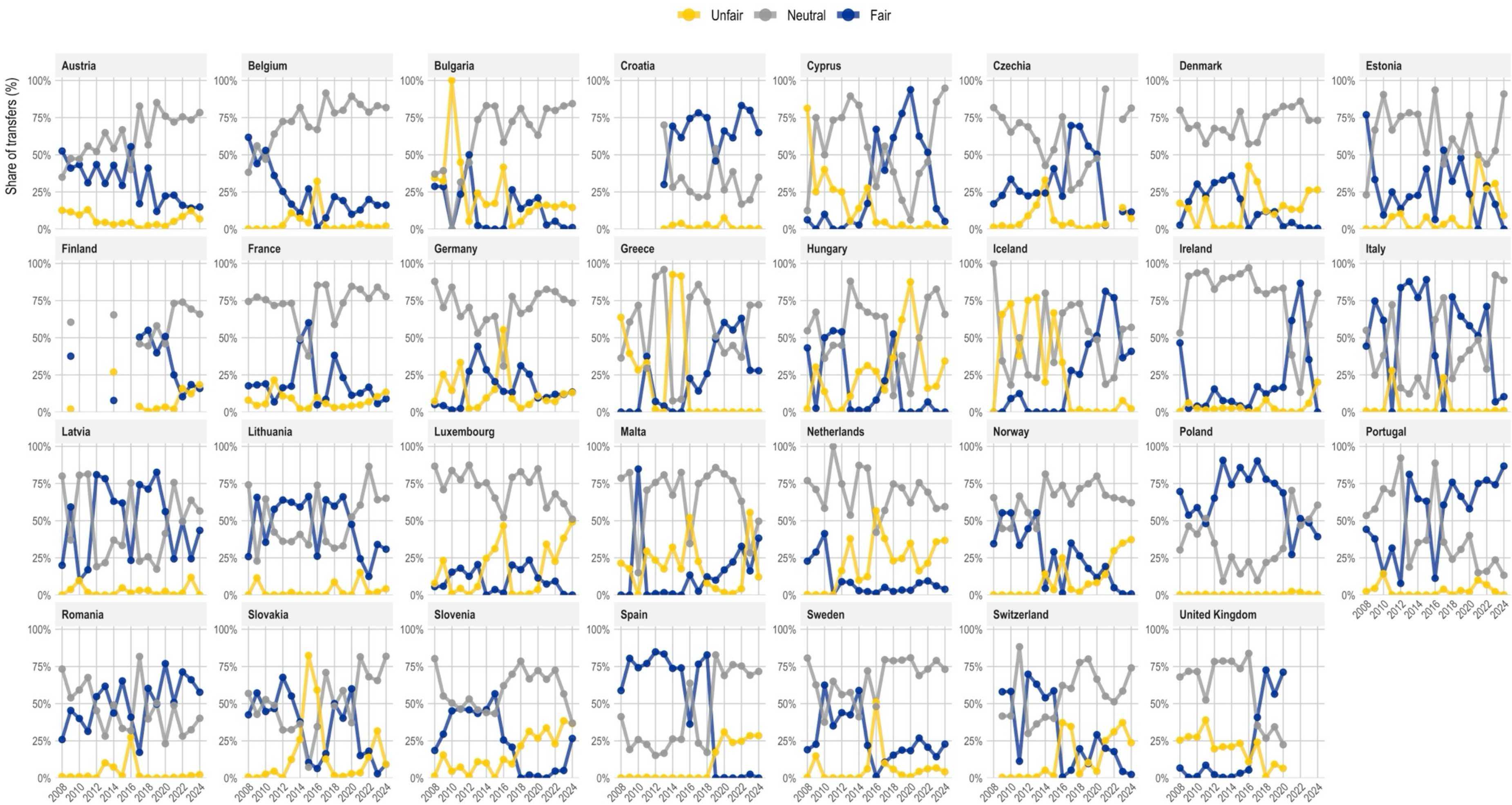

Note: Line plots of countries' Dublin transfers by fairness classification over time.

C.5: Dyad-level fairness outcomes over time by member state (aggregation reporting country)

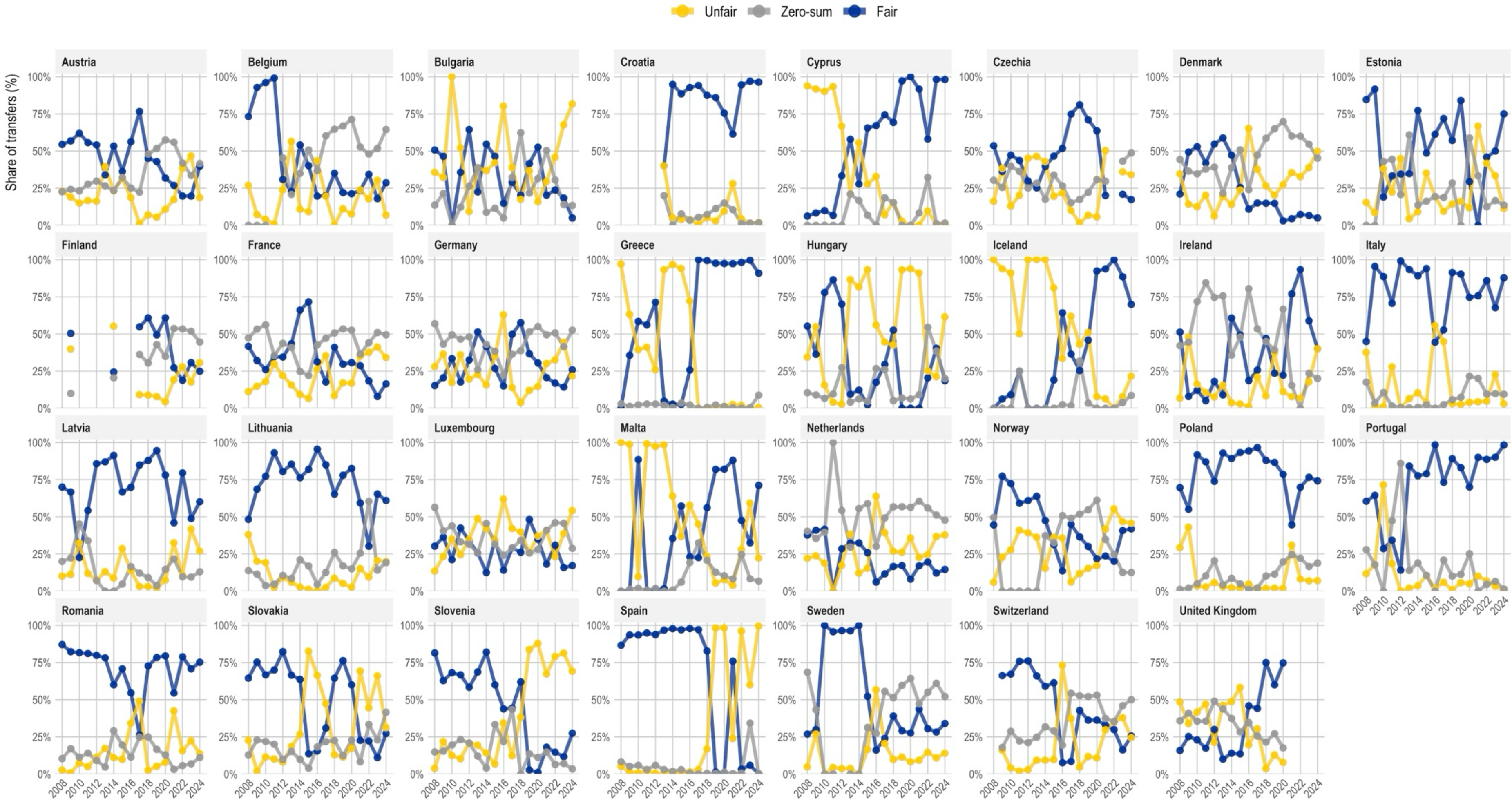

Note: Line plots of countries' Dublin transfers by fairness classification over time.

C.6: System-level fairness outcomes over time by member state (aggregation partner country)

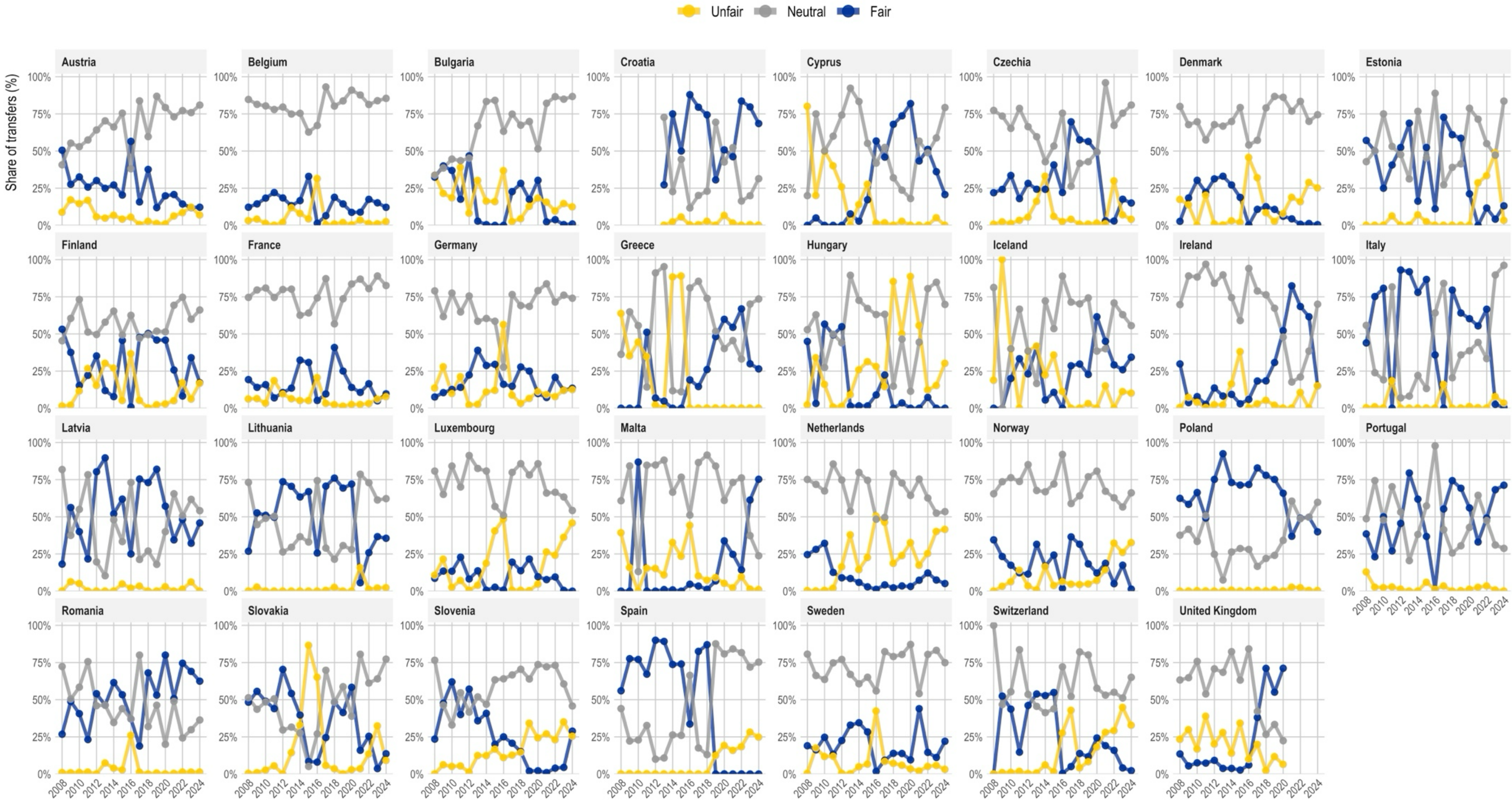

Note: Line plots of countries' Dublin transfers by fairness classification over time.

C.7: Dyad-level fairness outcomes over time by member state (aggregation partner country)

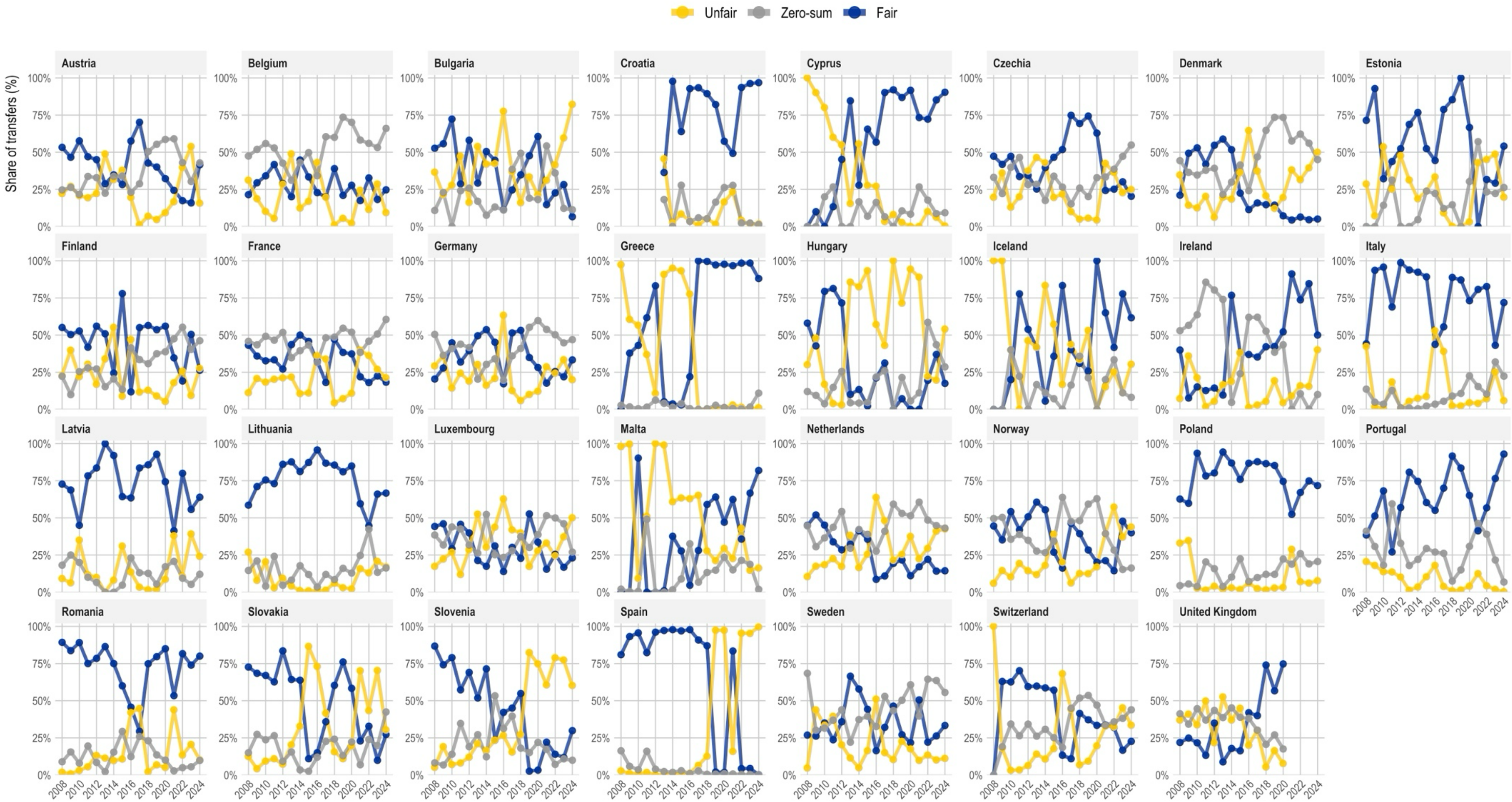

Note: Line plots of countries' Dublin transfers by fairness classification over time.

C.8: Choropleth of countries' fair contribution ratio (reporting country aggregation)

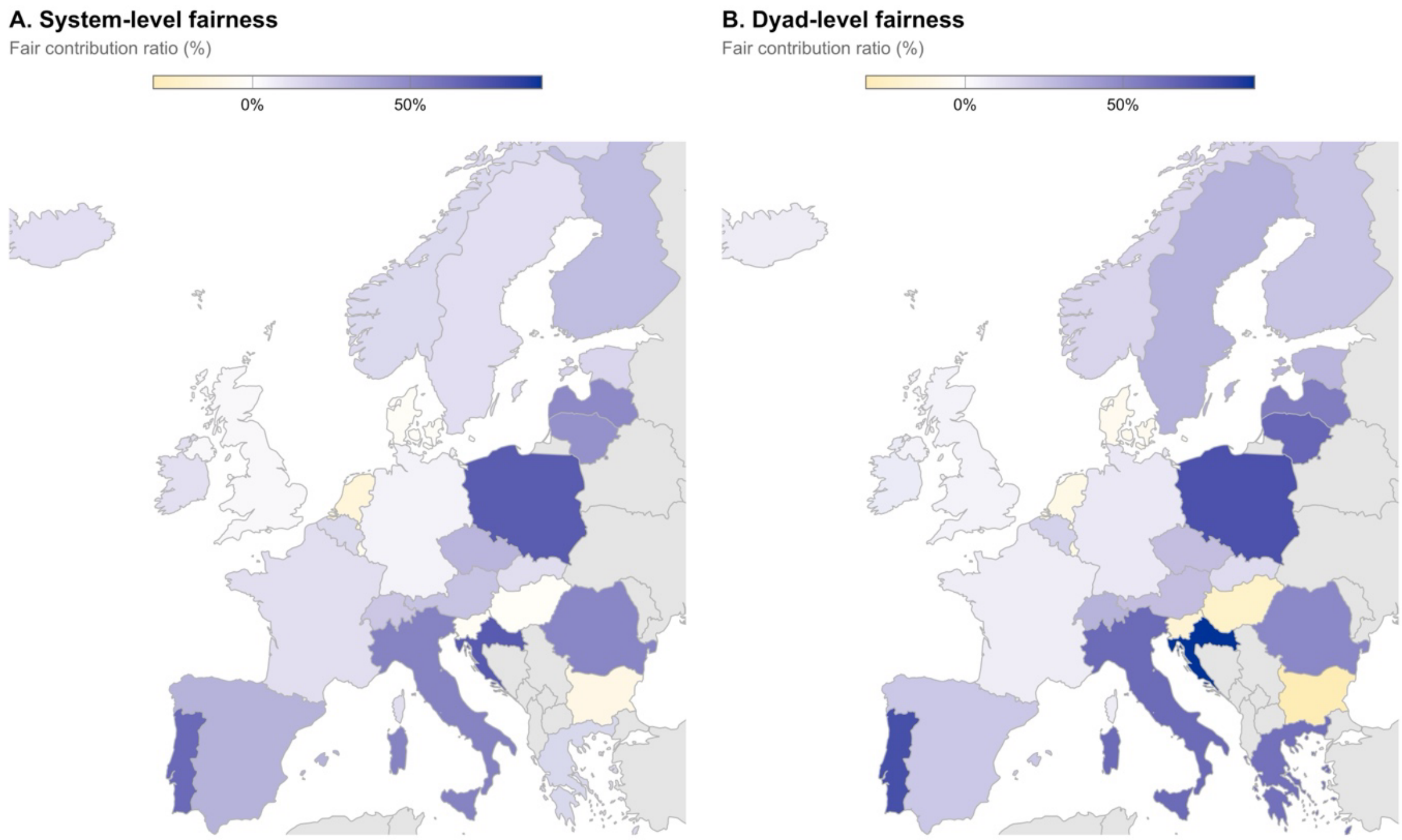

Note: Choropleth maps showing the fair contribution ratio of member states between 2008 and 2024. The ratio is the share of aggregated directional redistributive transfers divided through the total number of transfers. Aggregation based on the statistical figures provided by the reporting country.

C.9: Choropleth of countries' fair contribution ratio (partner country aggregation)

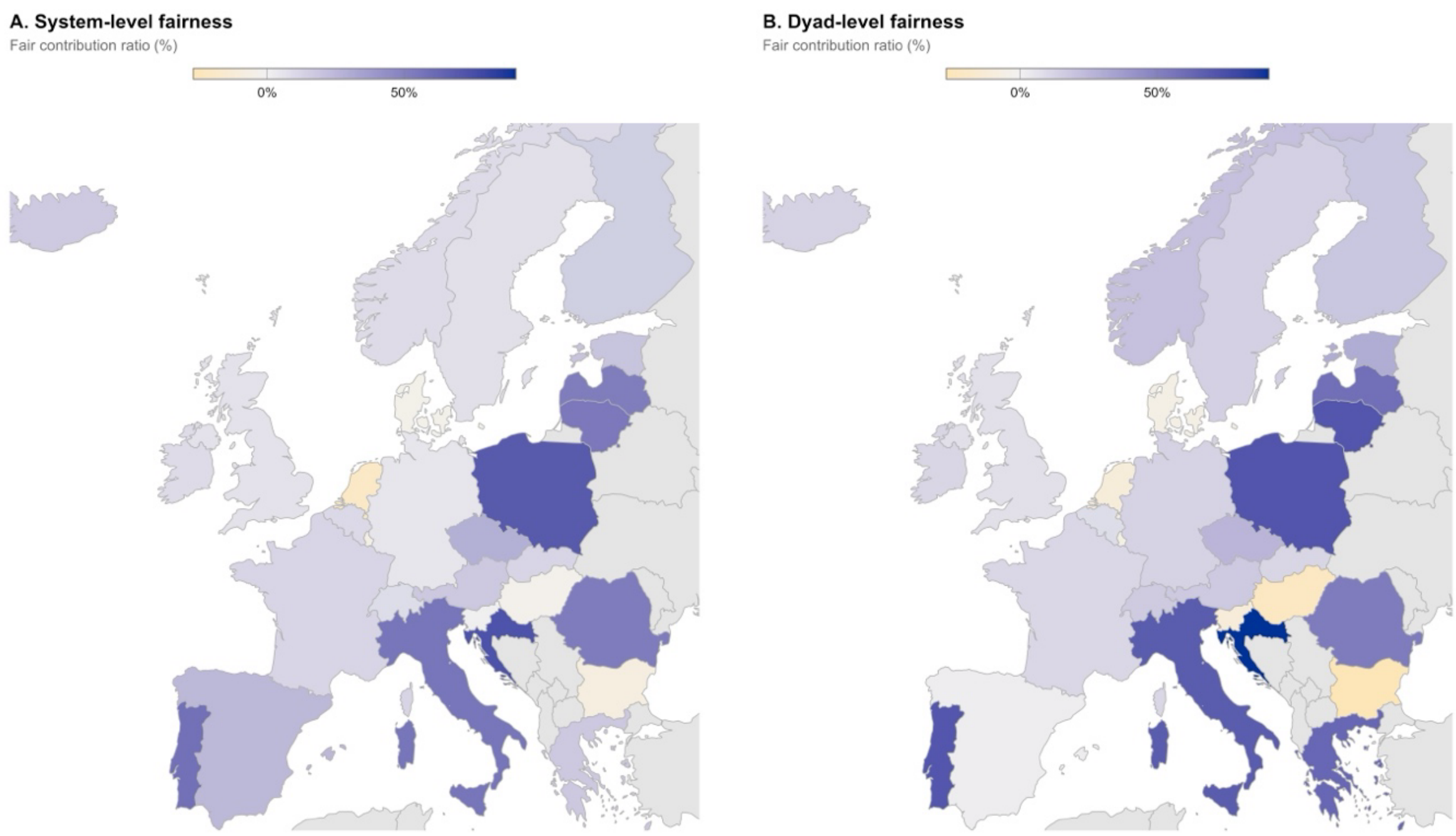

Note: Choropleth maps showing the fair contribution ratio of member states between 2008 and 2024. The ratio is the share of aggregated directional redistributive transfers divided through the total number of transfers. Aggregation based on the statistical reporting of the partner countries.

C.10: Share of Dublin transfers by fairness outcome (outgoing vs. incoming statistics)

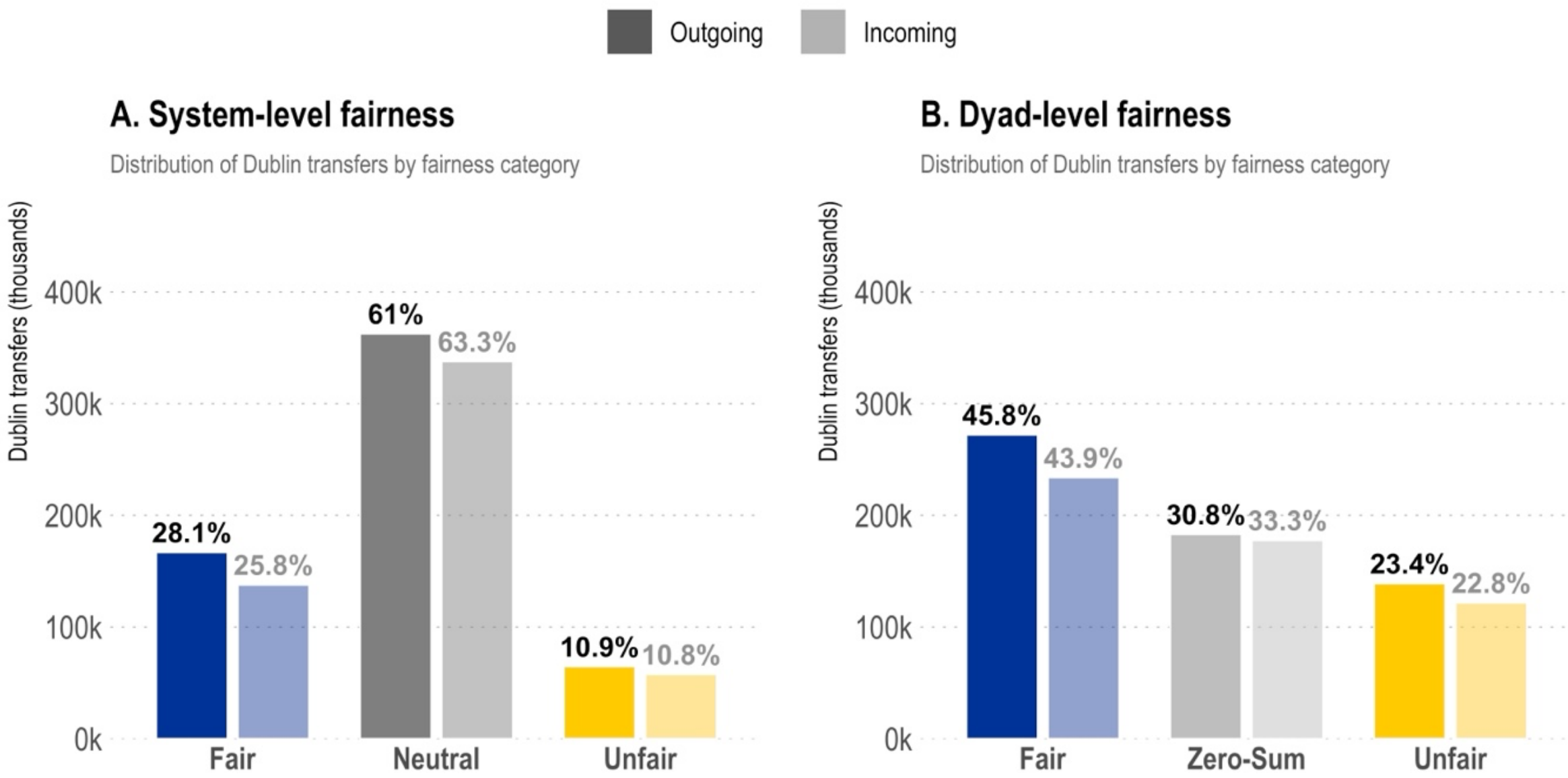

Note: The same plot as in Figure 3 in the manuscript but calculated separately based on the statistics of outgoing transfers (dark bars) and incoming statistics (light bars).

C.11: Share of Dublin transfers by fairness category excluding legal suspensions

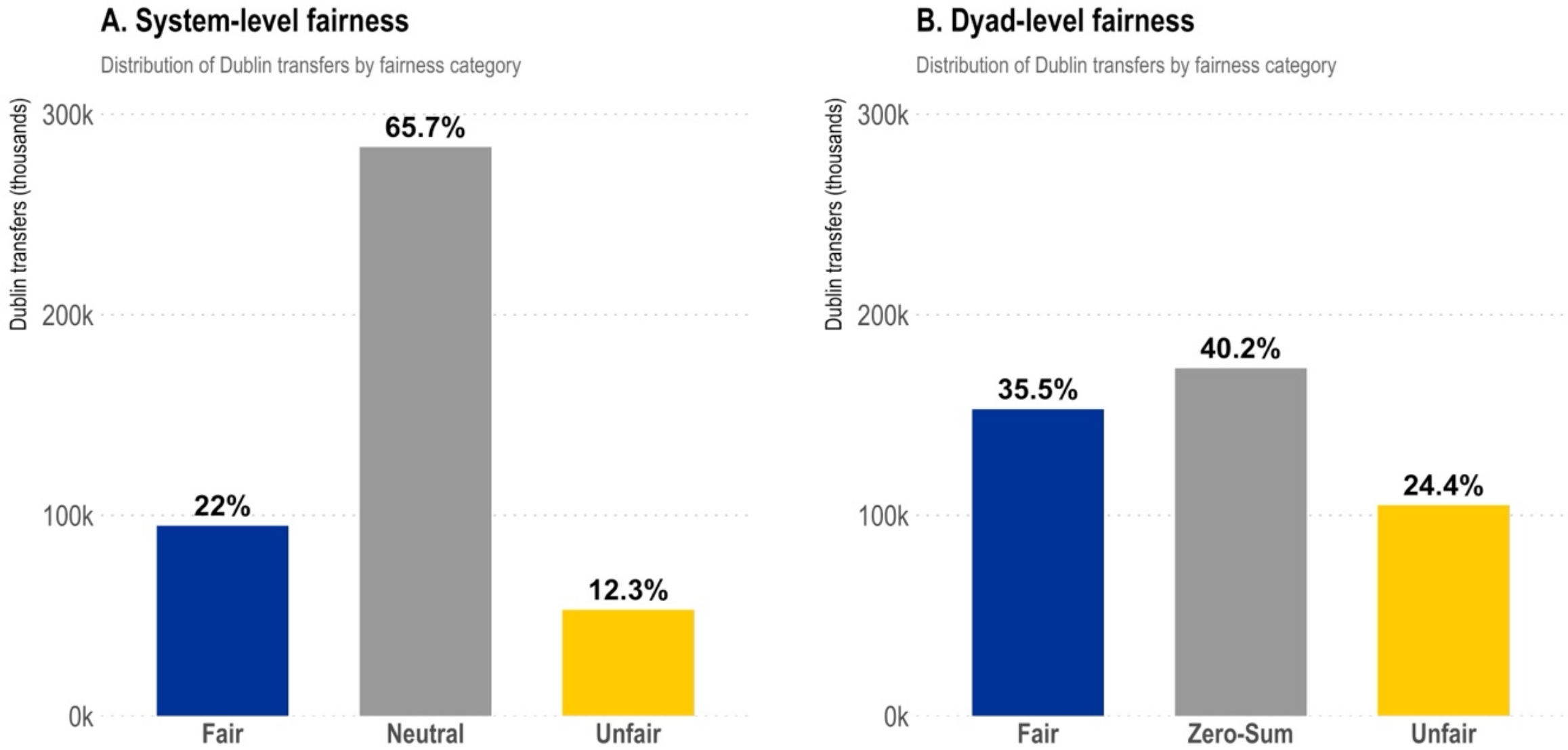

Note: Fairness classification of Dublin transfers based on the subsample of dyad-years without suspension. For the list of suspensions see Appendix II above.

C.12: Fairness simulation based on Dublin requests and positive decisions

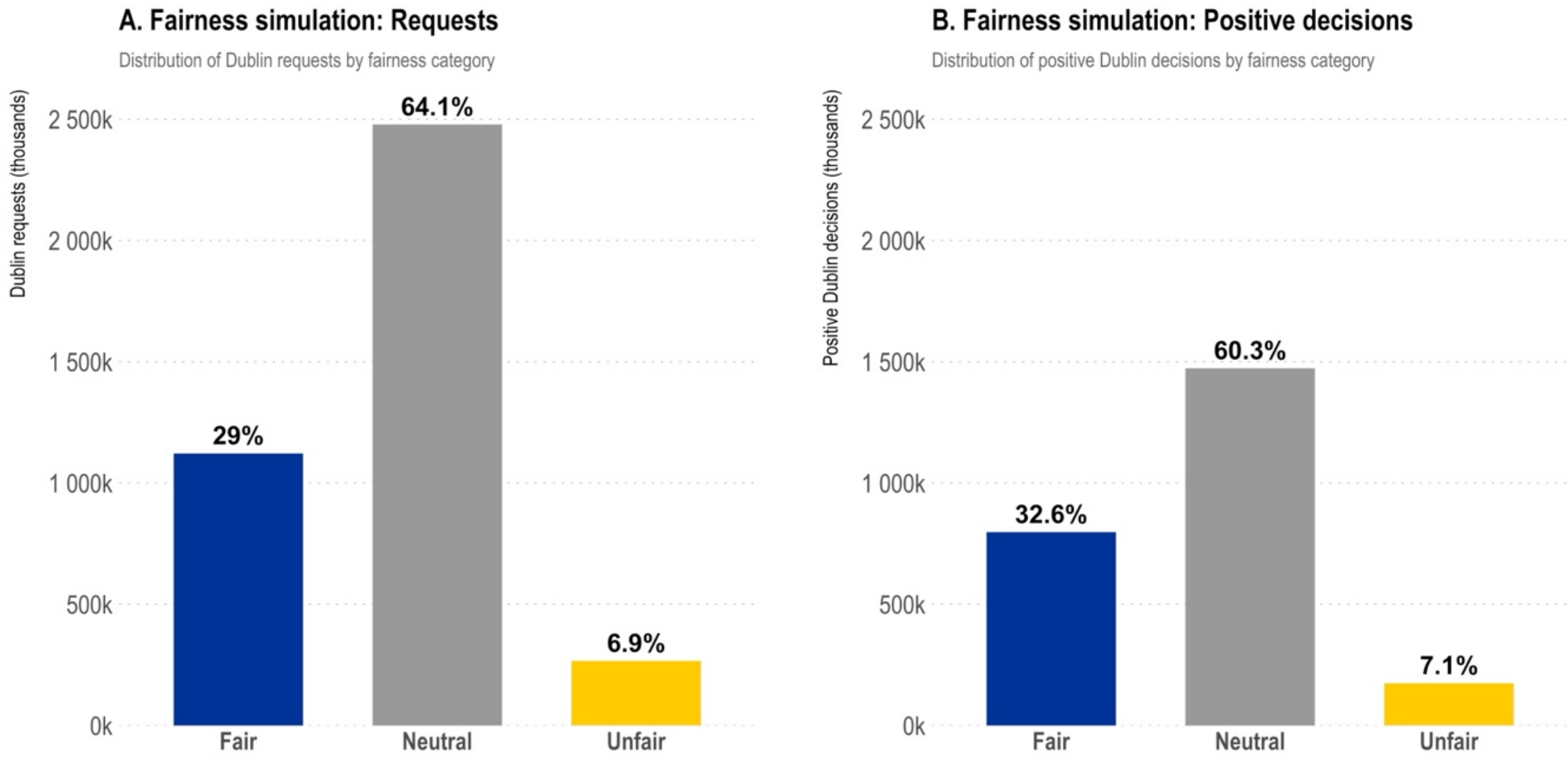

Note: Fairness calculation of hypothetical Dublin transfers that would have materialised in case of a fully effective Dublin system where all requests and positive decisions of those requests would have resulted in actual transfers.

C.13: Fairness simulation with Dublin requests and positive decisions (Eurodac hits)

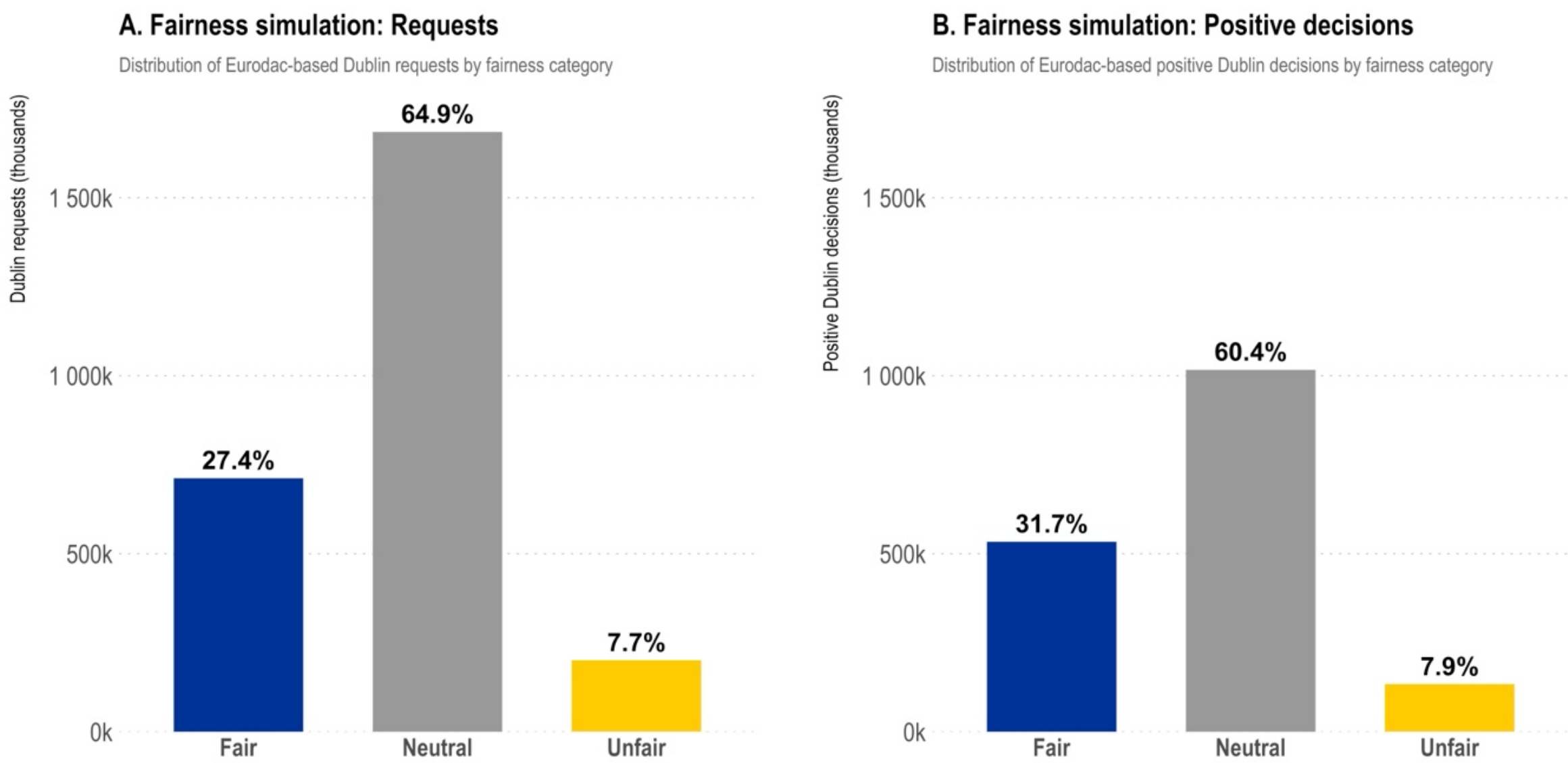

Note: The statistical data on Eurodac hits is based on the Eurostat datasets migr_dubredaco (https://doi.org/10.2908/MIGR_DUBREDACO), migr_dubredaci (https://doi.org/10.2908/MIGR_DUBREDACI), migr_dubdedaco (https://doi.org/10.2908/MIGR_DUBDEDACO) and migr_dubdedaci (https://doi.org/10.2908/MIGR_DUBDEDACI).

C.14: Top 10 countries using the Sovereignty Clause, 2014-2024

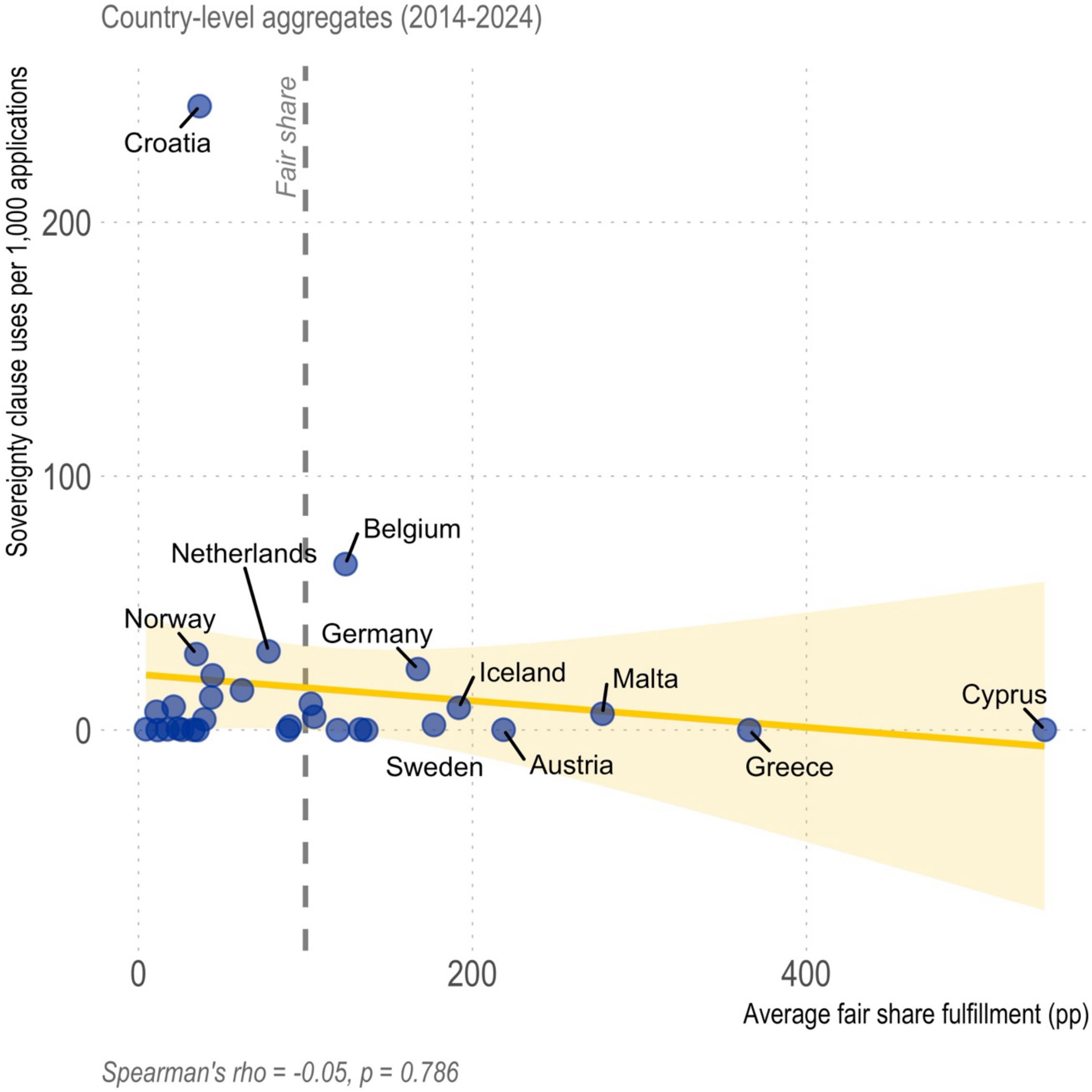

Note: The figure displays the relationship between countries' average fair-share fulfilment (percentage points above or below fair share) and their sovereignty clause usage rate (invocations per 1,000 asylum applications) for 2014-2024. The vertical line marks the fair-share threshold (100pp). Countries to the right exceed their fair share; those to the left fall short.
